# *Lactobacillus plantarum* ameliorates NASH-related inflammation by upregulating l-arginine production

**DOI:** 10.1038/s12276-023-01102-0

**Published:** 2023-11-01

**Authors:** Dong Yun Kim, Jun Yong Park, Heon Yung Gee

**Affiliations:** 1 Department of Pharmacology, Yonsei University College of Medicine, Seoul, Republic of South Korea; 2 Division of Gastroenterology, Department of Internal Medicine, Yonsei University College of Medicine, Seoul, Republic of South Korea; 3 Yonsei Liver Center, Severance Hospital, Seoul, Republic of South Korea; 4 Graduate School of Medical Science, Brain Korea 21 Project, Yonsei University College of Medicine, Seoul, Republic of South Korea; 5 Woo Choo Lee Institute for Precision Drug Development, Seoul, Republic of South Korea

**Keywords:** Non-alcoholic steatohepatitis, Biological therapy

## Abstract

*Lactobacillus* is a probiotic with therapeutic potential for several diseases, including liver disease. However, the therapeutic effect of *L. plantarum* against nonalcoholic steatohepatitis (NASH) and its underlying mechanisms remain unelucidated. Therefore, we delineated the *L. plantarum-*mediated NASH regulation in a mouse model to understand its therapeutic effect. We used a choline-deficient high-fat diet (CD-HFD)-induced murine model that recapitulated the critical features of human metabolic syndrome and investigated the effect of *L. plantarum* on NASH pathogenesis using transcriptomic, metagenomic, and immunohistochemistry analyses. Validation experiments were performed using liver organoids and a murine model fed a methionine-choline-deficient (MCD) diet. *L. plantarum* treatment in mice significantly decreased liver inflammation and improved metabolic phenotypes, such as insulin tolerance and the hepatic lipid content, compared with those in the vehicle group. RNA-sequencing analysis revealed that *L. plantarum* treatment significantly downregulated inflammation-related pathways. Shotgun metagenomic analysis revealed that L-arginine biosynthesis-related microbial genes were significantly upregulated in the *L. plantarum* group. We also confirmed the elevated arginine levels in the serum of the *L. plantarum* group. We further used liver organoids and mice fed an MCD diet to demonstrate that L-arginine alone was sufficient to alleviate liver inflammation. Our data revealed a novel and counterintuitive therapeutic effect of *L. plantarum* on alleviating NASH-related liver inflammation by increasing circulating L-arginine.

## Revolutionary Breakthrough: L. Plantarum Probiotic Alleviates Liver Inflammation

*Lactobacillus plantarum*, a beneficial gut bacteria, shows potential in treating non-alcoholic fatty liver disease (NAFLD) by increasing L-arginine levels and reducing liver inflammation. Researchers used a choline-deficient high-fat diet-induced NAFLD mouse model to study the effects of *L. plantarum* treatment. Results showed that *L. plantarum* administration ameliorated liver inflammation and enhanced L-arginine biosynthesis in the intestine. Furthermore, L-arginine treatment also improved liver inflammation in methionine-choline deficient-induced NAFLD mice. These findings suggest that *L. plantarum* could be a promising therapeutic strategy for NAFLD by increasing L-arginine levels and modulating gut microbiota, improving intestinal barrier integrity, and attenuating inflammation.

## Introduction

Nonalcoholic fatty liver disease (NAFLD) is the most common cause of chronic liver disease worldwide, affecting approximately one-quarter of the adult population^1^. NAFLD is characterized by triglyceride accumulation in over 5% of hepatocytes without excessive alcohol consumption and the onset of other secondary causes of hepatic steatosis^2,3^. The different forms of NAFLD include conditions such as nonalcoholic fatty liver (NAFL), nonalcoholic steatohepatitis (NASH), liver cirrhosis (LC), and hepatocellular carcinoma (HCC).

The ability of NASH to progress to LC and HCC has made it the second leading indication for liver transplantation in the USA, and it will likely top the list within a decade^4,5^. NASH, with its growing impact on the global health burden, is a significant research target for developing treatments; however, these attempts have been unsuccessful to date. Currently, only lifestyle interventions such as exercise and weight loss have been recommended to manage NASH^6,7^.

With the development of the “gut–liver axis” concept, comprehensive investigations are ongoing to decode the intestine–liver interactions. Increasing evidence indicates that the gut microbiota is an environmental factor affecting all histological components of NAFLD: hepatic steatosis, inflammation, and fibrosis^8^. Concordantly, several studies have suggested that probiotics, which confer health benefits directly to the host or indirectly by altering intestinal microbiome diversity^9^, could be a promising agent for treating NASH. Several putative mechanisms of the gut microbiome can affect NAFLD. One such mechanism involves bacterial proteins functioning as ligands for G protein-coupled receptors^10^, modulating the gut–liver axis through intestinal farnesoid X receptor signaling by releasing fibroblast growth factor 19, a gut-derived hormone that affects lipid and glucose metabolism and bile acid synthesis^11^. Another suggested mechanism involves their ability to intensify gut barrier function^8^. *Lactobacillus plantarum*, a species of beneficial gut lactic acid bacteria, is a safe and effective dietary supplement with specific favorable immune-regulating properties^12^. The effects of *L. plantarum* include maintaining gut homeostasis^13^ by restoring gut barrier function^14^, regulating the immune response^15^, and reducing liver enzymes^16^. The many beneficial effects of *L. plantarum* establish it as a potential candidate probiotic for NASH treatment^17^. However, the role of *L. plantarum* and its underlying mechanisms in treating NASH remain unclear. Therefore, we aimed to clarify its effect on NASH and to reveal its mechanism of action. Notably, we investigated whether *L. plantarum* could alleviate steatohepatitis and the extent and mechanisms by which it could affect NASH.

## Materials and methods

### Animal models

**Fig. 1:**
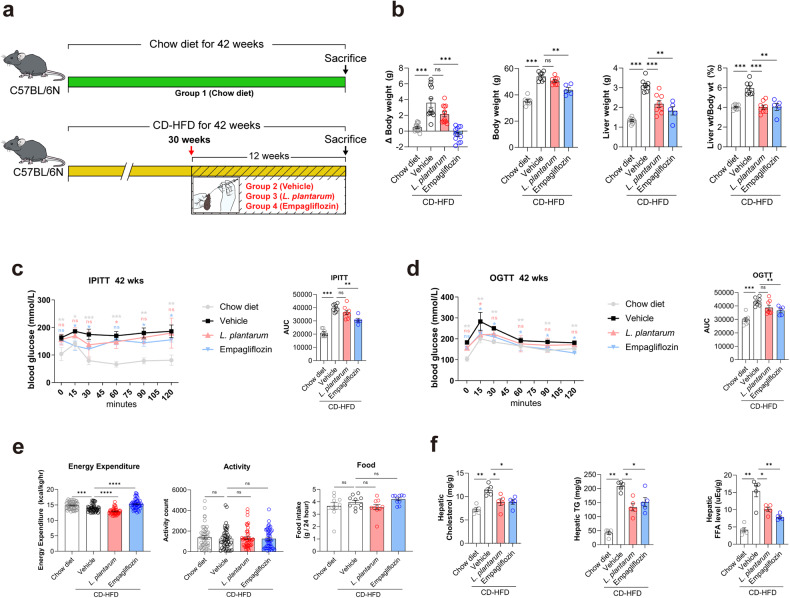
*L. plantarum* treatment improves the metabolic phenotypes associated with NAFLD. **a** A schematic of the treatment procedure. **b** Changes in body weight, liver weight, and the liver-to-body weight ratio. **c**, **d** Changes in insulin sensitivity. The insulin tolerance test (**c**) and oral glucose tolerance test (**d**) were performed at 42 weeks. **e** Energy expenditures, activity levels, and food intake were measured using metabolic cages (*n* = 3–4 per group). **f** Hepatic lipid levels. *P < 0.05, ***P < 0.001, and ****P < 0.0001 compared with the vehicle-treated CD-HFD mice using ANOVA and the post hoc Tukey test. CD-HFD choline-deficient high-fat diet, IPITT intraperitoneal insulin tolerance test, OGTT oral glucose tolerance test.

Six-week-old male C57BL/6 N mice were purchased from Orient Bio (Sungnam, South Korea). The mice had free access to diet and water; their ambient temperature was maintained at 23 ± 2 °C, the humidity was 60% ± 10%, and they were kept on a 12-h light/dark cycle. After seven days of adaptation, all mice except the chow diet group (control) were fed a choline-deficient high-fat diet (CD-HFD) for 30 weeks to induce NASH. After NASH induction, the mice were randomly assigned to one of the following three groups, with eight mice in each group: CD-HFD vehicle-, CD-HFD *L. plantarum*-, or CD-HFD empagliflozin-treated groups (Fig. 1a). *L. plantarum* (1 × 10^9^ CFU) and empagliflozin (10 mg/kg body weight of the mice) were given once daily by oral gavage to the respective mice for 12 weeks. Empagliflozin has a positive effect on NAFLD mouse models; therefore, it was also included in the treatment to better evaluate the effect of *L. plantarum*. The chow diet and the CD-HFD plus vehicle group received the same volume of PBS orally for 12 weeks, after which the mice were anesthetized and killed, and blood was collected via heart puncture. Tissues were harvested and either snap-frozen in liquid nitrogen and stored at −70 °C or fixed in formalin and embedded in paraffin. All animal-related procedures were approved by the Animal Care and Use Committee of the Yonsei University College of Medicine.

### Drugs and diets

Empagliflozin was purchased from Boehringer Ingelheim Pharma GmbH Co. (KG, Biberach a der Riss, Germany), and *L. plantarum* was supplied by Ildong Bioscience Co., Ltd. l-Arginine was purchased from Sigma‒Aldrich (#A5131, USA). The mice were fed a chow diet, CD-HFD (#D05010402, Research Diet, NJ, USA), or methionine–choline-deficient (MCD) diet (#A02082002BR, Research Diet, NJ, USA). The chow diet contained 22% protein, 6% fat, and 47% carbohydrate. The D05010402 CD-HFD contained no choline chloride; it had 22.6% protein, 23.5% fat, accounting for 43% of the energy in the diet, and 5.4% fiber. The A02082002BR MCD diet contained 16.9% protein, 9.9% fat, and 64.9% carbohydrates.

### Human liver transcriptome and blood samples

A total of 33 liver samples from Severance Hospital were included in this study. These patients were selected based on the isolation of sufficient high-quality RNA for sequencing. Available samples consisted of 28 patients with NAFLD and five control participants from among liver transplantation donors. Liver samples were obtained by liver biopsy (NAFLD patients) or liver transplant operation (healthy donors). Additionally, serum levels of l-arginine were measured using an ELISA kit (ImmuSmol, Bordeaux, FRANCE, #IS I-0400) in individuals for whom serum samples were available. The collection and use of samples for this study were approved by the Severance Hospital Institutional Review Board (institutional review board number: #4-2018-0537) and conducted following the principles of the Declaration of Helsinki.

### High-throughput RNA-seq analysis

Mouse liver and ileum tissue samples were lysed using TRIzol (Sigma‒Aldrich), and mRNA was extracted with an Allprep DNA/RNA Micro kit (Qiagen). The concentrations and quality of the samples were assessed using an Agilent Pico 6000 kit with a Bioanalyzer 2100 (Agilent). After cDNA synthesis, the library was constructed, pooled, and sequenced on a NovaSeq System (Illumina). Libraries were constructed with a TruSeq RNA Library Sample Prep kit (Illumina; San Diego, CA, USA), and the enriched library was sequenced on an Illumina HiSeq 2500 system. CLC Genomics Workbench 9.5.3 software (Qiagen, Germany) was used to map the reads to the mouse genome (NCBI GRCm38/mm10) or human genome (NCBI GRCh38/Hg38) with default parameters. The number of raw read counts was transformed to variance transformed data (using “DESeq” in the R package^18^) for the transcriptomic analysis. The up- or downregulated differentially expressed genes (DEGs) were estimated by the previously reported method and significance criteria (adjusted *P* value < 0.05, |Log2 (fold change) |≥1) (using “DESeq” in the R package^18^). Gene set enrichment analysis was performed for Gene Ontology (GO), hallmark gene sets, or Kyoto Encyclopedia of Genes and Genomes (KEGG) gene sets using GSEA v.4.3.1 for Windows from the Molecular Signatures Database (MSigDB; http://www.gsea-msigdb.org/gsea/msigdb/index.jsp).

### Culture of mouse liver organoids and Kupffer cells

Kupffer cells were grown in DMEM supplemented with 10% FBS and 1% penicillin/streptomycin (Thermo Fisher Scientific) at 37 °C in a humidified 5% CO_2_ atmosphere. Mouse liver organoids were purchased from STEMCELL^TM^ (USA, #70932). Liver organoids were cultured in HepatiCult^TM^ Organoid Growth Medium (#06030, STEMCELL^TM^, USA) embedded in a 40-μL droplet of Matrigel (#356231, Corning, USA) in 24-well plates. The liver organoids were differentiated and maintained for two weeks in a Transwell system (#3460, Corning, USA), and the medium was changed every two days. When the liver organoids were fully differentiated, a Transwell containing confluent Kupffer cells was inserted over each organoid. The cells were then treated with lipopolysaccharide (LPS, 100 ng/ml; Sigma‒Aldrich) for 24 h to induce a NASH microenvironment. Finally, the cells were treated with either DMSO or l-arginine (1 mM) in a Transwell plate for 24 h.

### Immunofluorescence analysis

The liver organoids were fixed by immersing them in 4% formaldehyde at 4 °C overnight for immunofluorescence. The fixed liver organoids were washed three to four times in PBS and incubated in a blocking buffer containing 10% goat serum and 0.5% Triton X-100 in PBS for 1 h at room temperature. For staining, 1:100 and 1:1000 dilutions of primary and fluorophore-tagged secondary antibodies, respectively, were used (Supplementary Table 1). Alexa 568- and Alexa 647-conjugated secondary antibodies and 4′,6-diamidino-2-phenylindole dihydrochloride were obtained from Invitrogen. Confocal images were obtained with a Carl Zeiss LSM780 instrument, and ZEN software was used for image processing.

### Real-time PCR analysis

For real-time quantitative PCR, RNA samples were isolated from the mouse liver tissues, Kupffer cells and liver organoids with RiboEx (GeneAll Biotechnology) and reverse-transcribed with an iScript cDNA Synthesis Kit (Bio-Rad). The samples were assayed with SYBR Green ready master mix (#4309155, Fisher Scientific, USA); primers used for this assay are listed in Supplementary Table 2. Real-time PCR was performed on a StepOnePlus RT‒PCR system (Applied Biosystems, Waltham, MA, USA). The relative mRNA expression levels were calculated via the comparative threshold cycle (Ct) method, and glyceraldehyde 3-phosphate dehydrogenase (*GAPDH*) was used as the housekeeping control.

### Metagenome shotgun sequencing

Genomic DNA was isolated from the murine cecal contents using a QIAamp DNA Stool MiniKit (Qiagen, Germany). The quantity and quality of the isolated DNA samples were assessed using PicoGreen dsDNA quantitation reagent (Invitrogen, Carlsbad, CA, USA) and agarose gel electrophoresis, respectively. Each sequenced sample was prepared according to the Illumina protocols. Briefly, 100 ng of genomic DNA was fragmented into 150-bp inserts using a Covaris-focused ultrasonicator (Covaris, Inc., Woburn, MA, USA). The fragmented DNA was blunt-ended and phosphorylated. After repair, the appropriate library size was selected using different proportions of sample purification beads. A single “A” base was ligated to the 30 ends of the fragmented DNA, followed by ligation of the Illumina adapters. The final ligated product was quantified by qPCR according to the qPCR Quantification Protocol Guide, and the quality was assessed using a 2200 TapeStation (Agilent Technologies, Palo Alto, CA, USA). Sequencing was performed on a HiSeq 4000 platform (Illumina, San Diego, CA, USA). Raw data from the whole metagenome shotgun sequencing were processed through read-level quality control (KneadData), taxonomic profiling (MetaPhlAn), functional profiling (HUMAnN), and strain profiling (StrainPhlAn) via the “bioBakery workflows” (http://huttenhower.sph.harvard.edu/biobakery_workflows).

### Microbiota composition analyses

Pyrosequencing of 16 S rDNA amplicons was used to analyze the microbiota composition, and qPCR was performed according to a previous study^19^. Following specimen collection, bulk genomic DNA was prepared using robust extraction protocols. The collected DNA was subjected to broad-range PCR amplification using primers that amplify all bacterial 16 S rRNA genes. PCR amplicons were sequenced using next-generation sequencing platforms. The resulting sequence datasets were quality-filtered, and each sequence was assigned a taxonomic classification using phylogenetic analysis.

### Biochemical parameters

Blood samples were collected by heart puncture into plasma separation tubes with lithium heparin (Biosciences, Franklin Lakes, NJ, USA), followed by centrifugation at 2000 × *g* for 15 min at 4 °C. Enzymatic colorimetric assay kits for aspartate aminotransferase (AST; Fujifilm DRI-CHEM SLIDE, Tokyo, Japan), alanine transaminase (ALT; Fujifilm DRI-CHEM SLIDE), total cholesterol (Fujifilm DRI-CHEM SLIDE), and triglycerides (Fujifilm DRI-CHEM SLIDE) were used to determine plasma levels on a DRI-CHEM NX700 (Fujifilm).

### Histological analysis

Liver histological analyses were performed using standard methods on paraffin-embedded sections with hematoxylin and eosin (H&E) staining. Fibrosis was assessed in paraffin-embedded sections with Sirius red staining using established methods^20^. The liver histology results were evaluated in a blinded manner by an expert liver pathologist unaware of the dietary conditions of the experimental mice. Histology was assessed using the NASH Clinical Research Network (CRN) and fatty liver inhibition of progression consortium criteria^21^.

### Quantification of hepatic lipid profiles

After homogenization, total cholesterol, triglyceride, and free fatty acid (FFA) levels in liver tissues were measured using the Total Cholesterol and Cholesteryl Ester Colorimetric/Fluorometric Assay (#K603; BioVision, Milpitas, CA, USA), Triglycerides Quantification (#K622; BioVision, Milpitas, CA, USA), and Free Fatty Acid Quantification Colorimetric/Fluorometric Kits (#K612; BioVision, Milpitas, CA, USA), respectively, according to the relevant manufacturer’s instructions.

### Glucose and insulin tolerance tests

Before killing, the mice were fasted overnight, and their baseline blood glucose levels were measured in tail-vein blood using an Accu-Chek Compact Plus glucometer (Roche Diagnostics). Subsequently, 1 mg dextrose/g body weight in sterile PBS was injected intraperitoneally, and blood glucose levels were measured after 15, 30, 60, 90, and 120 min using established protocols^21^. For insulin tolerance testing, mice were fasted for 6 h, and regular insulin (0.75 U/kg body weight) was intraperitoneally injected using an established methodology^21^. Blood glucose levels were measured before and 15, 30, 60, 90, and 120 min after insulin injection. The glucose and insulin tolerance tests were performed on the same mice for several days in succession.

### Immunohistochemical analysis of liver tissue

Immunohistochemistry (IHC) was conducted by deparaffinizing paraffin-embedded sections in xylene and rehydrating them using a decreasing gradient of ethanol. Antigen retrieval was performed by incubation in 10 mM sodium citrate buffer (pH 6.0), after which sections were incubated overnight at 4 °C with primary antibodies (Supplementary Table 1). Then, the sections were incubated with the appropriate biotinylated secondary antibody and treated with freshly prepared 3,3′-diaminobenzidine substrate. The sections were lightly counterstained with hematoxylin and mounted. Images were processed and analyzed using ImageJ and Adobe Illustrator software (version: 26.5).

### FITC-dextran permeability assay

Intestinal permeability was assessed by orally administering FITC-dextran 4000 (FD4K) (Sigma–Aldrich, St. Louis, MO, USA). After fasting for 4 h, the mice were subsequently gavage-fed 60 mg/100 g body weight FITC-dextran solution. Mice were killed 4 h later, and the FITC concentration in systemic serum was measured. FITC-dextran was measured in duplicate by fluorometry with excitation and emission values of 490 nm and 530 nm, respectively, using a Cytofluor 2300 fluorometer (Millipore). Serial dilutions of FITC-dextran in PBS were used to prepare a standard curve.

### Culture of Caco-2 cells and immunoblotting analysis

Caco-2 cell lines were kindly provided by Prof. Jun Yong Park, Yonsei University of South Korea. They were cultured in Dulbecco’s modified Eagle’s medium (DMEM) supplemented with 20% fetal bovine serum (FBS) and 1% penicillin/streptomycin (Thermo Fisher Scientific). Total protein was extracted from the Caco-2 cells using lysis buffer and clarified by centrifugation. The protein content of the supernatants was separated by 10% SDS‒PAGE in a Bio-Rad system and transferred onto a polyvinylidene fluoride membrane (Bio-Rad). After blocking with 5% BSA for 30 min, the membranes were incubated overnight at 4 °C with the appropriate primary antibodies for #3195 E-cadherin (CST), #87402 occludin (Novus), and #ab20272 β-actin (Abcam). They were then incubated with horseradish peroxidase (HRP)-conjugated secondary antibody for 1 h (Supplementary Table 1). Bands were detected using Clarity Western ECL.

### Serum endotoxin level analysis

Serum was diluted at a 1:10 ratio with pyrogen-free water. Endotoxin measurements were performed in pyrogen-free glass tubes, Eppendorf tubes, and plates. According to the manufacturer’s instructions, endotoxin levels were determined using a Pierce LAL Chromogenic Endotoxin Quantitation Kit (Thermo Fisher Scientific, Waltham, MA, USA).

### ELISA for serum l-arginine analyses

Serum levels of l-arginine were quantified using enzyme-linked immunosorbent assay (ELISA) kits (ImmuSmol, Bordeaux, FRANCE, #IS I-0400) according to the manufacturer’s instructions. Briefly, the standards, controls, and serum samples were diluted to a 1:10 ratio and placed in 96-well plates. Arginine antiserum was added to the wells and incubated overnight at 4 °C. Then, the plates were washed three times with wash buffer composed of 0.05% P1379 Tween 20 (Sigma‒Aldrich) in PBS, with a pH of 7.4. The enzyme conjugate was added to the wells and incubated at room temperature for 30 min. Then, the plates were washed four times with washing buffer, and the stop solution was added to each well. The optical density of the resulting solution was measured at 450 nm.

### Metabolic analysis

The mice were individually housed in an eight-chamber, open-circuit Oxymax/Comprehensive Lab Animal Monitoring System (Columbus Instruments, Columbus, OH, USA) to measure their metabolic rates. After one day of acclimation, each mouse was assessed for oxygen consumption, carbon dioxide production, activity, and food intake for 72 h with access to diet and water ad libitum. Heat production was calculated as:

$$(3.815+1.232\times {RER})\times {VO}2$$

where RER is the respiratory exchange ratio, which was calculated as VCO2 VO2^−1^.

### Statistical analysis

Statistical analyses used in this study included unpaired nonparametric t tests and Mann‒Whitney tests with Tukey’s multiple-comparison post hoc tests. *P* values < 0.05 were considered statistically significant. Statistical methods for comparing experimental groups are indicated in the figure legends. Significant differences between two groups are denoted by asterisks (*P < 0.05; **P < 0.01; and ***P < 0.001). All statistical analyses were conducted using SAS 9.4 software (SAS Institute, Cary, NC, USA). Data visualization and analysis were performed using BioRender (www.BioRender.com) and GraphPad Prism version 9.4.1 for Windows (GraphPad Software, San Diego, California, USA, www.graphpad.com).

## Results

### *L. plantarum* treatment ameliorates metabolic phenotypes associated with NAFLD

We observed that mice fed a CD-HFD for 42 weeks gained more weight with a higher rate of obesity than that of the chow diet-fed control mice (Fig. 1a, b). Empagliflozin, a sodium/glucose cotransporter inhibitor used for diabetes, was included in our investigation to compare to the treatment effect of *L. plantarum*, and empagliflozin significantly reduced total body weight in CD-HFD-fed mice (Fig. 1b). *L. plantarum* and empagliflozin treatments significantly decreased liver weights and the liver-to-body weight ratios compared to those of the vehicle group (Fig. 1b). Even though the CD-HFD led to greater insulin resistance and impaired glucose tolerance at 42 weeks compared to those of the chow diet, only empagliflozin significantly improved insulin resistance and glucose tolerance compared to vehicle. In contrast, *L. plantarum* did not substantially affect insulin resistance or glucose tolerance (Fig. 1c, d). In addition, only mice treated with empagliflozin expended more energy than mice treated with the vehicle, although none of the treatments demonstrated a statistically significant difference in activity count or food intake relative to the vehicle-treated mice (Supplementary Fig 1 and Fig. 1e). These results collectively indicated the ability of *L. plantarum* to relieve NAFLD-related metabolic phenotypes without affecting systemic energy metabolism.

### *L. plantarum* attenuates liver inflammation

**Fig. 2:**
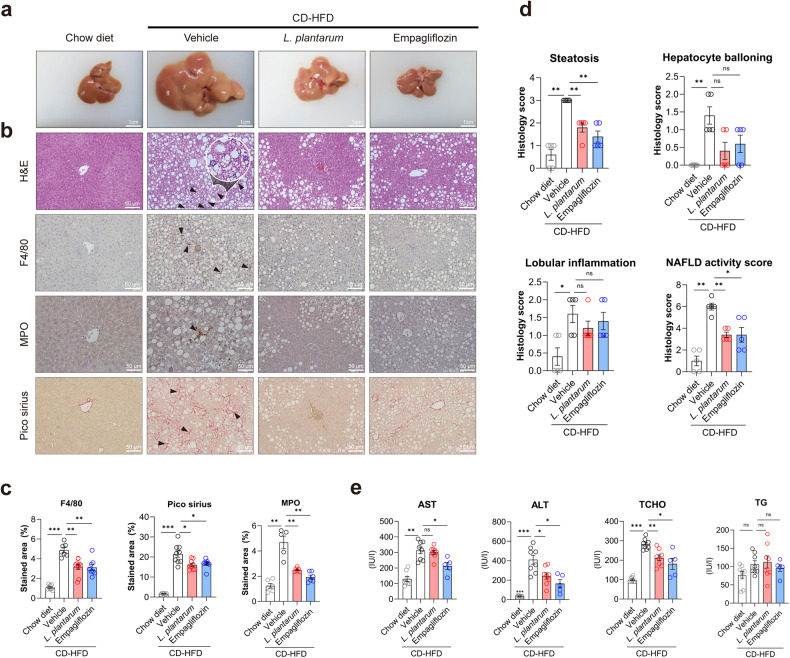
*L. plantarum* administration ameliorates inflammation in NAFLD. **a** Gross specimens of livers from the indicated groups. Scale bar, 1 cm. **b** Representative photomicrographs of liver sections stained with H&E and immunohistochemistry images of paraffin-embedded sections stained with F4/80, MPO, and Sirius red. Scale bar, 50 μm. **c** Quantification of the stained areas of **b**. **d** NAS scores. **e** Serum biochemical results. *P < 0.05, **P < 0.01, and ***P < 0.001 compared with the vehicle-treated CD-HFD mice, using ANOVA and the post hoc Tukey test. CD-HFD choline-deficient high-fat diet, MPO myeloperoxidase, AST aspartate aminotransferase, ALT alanine aminotransferase, TCHO total cholesterol, TG triglyceride.

The livers of *L. plantarum-* and empagliflozin-treated mice were smaller (Fig. 2a), possibly due to there being less fat (Fig. 1f), compared with the size of the control group livers. To further investigate the molecular pathways underlying the decrease in hepatic lipid accumulation observed in mice treated with *L. plantarum*, quantitative reverse transcription polymerase chain reaction (qRT‒PCR) analysis was performed. Our results confirmed the upregulation of fatty acid oxidation-related genes in the livers of *L. plantarum*-treated mice (Supplementary Fig. 2a–d). In addition, we analyzed GO gene sets using GSEA. Gene sets associated with cholesterol homeostasis were significantly enriched in mice treated with *L. plantarum* (Supplementary Fig. 2e). These findings suggest that *L. plantarum* treatment may contribute to the improvement in hepatic steatosis by influencing these molecular pathways. Hematoxylin-eosin (H&E) staining confirmed that CD-HFD-fed control mice developed steatohepatitis, which was characterized by steatosis, lobular inflammation, and hepatocellular ballooning (Fig. 2b). Immunohistochemistry of F4/80 and MPO, which label macrophages and neutrophils, respectively, revealed that the immune cell-positive areas were increased by the CD-HFD. In contrast, they were significantly reduced by *L. plantarum* or empagliflozin treatment (Fig. 2b, c). *L. plantarum* also significantly decreased the NAFLD activity score compared with that in the vehicle group (Fig. 2d). Biochemical analysis demonstrated that the serum level of ALT, a liver injury marker, was also significantly lower in *L. plantarum-*treated mice (Fig. 2e) than in the control group.

### Transcriptomic analysis reveals that *L. plantarum* reduces inflammatory pathways in the liver

**Fig. 3:**
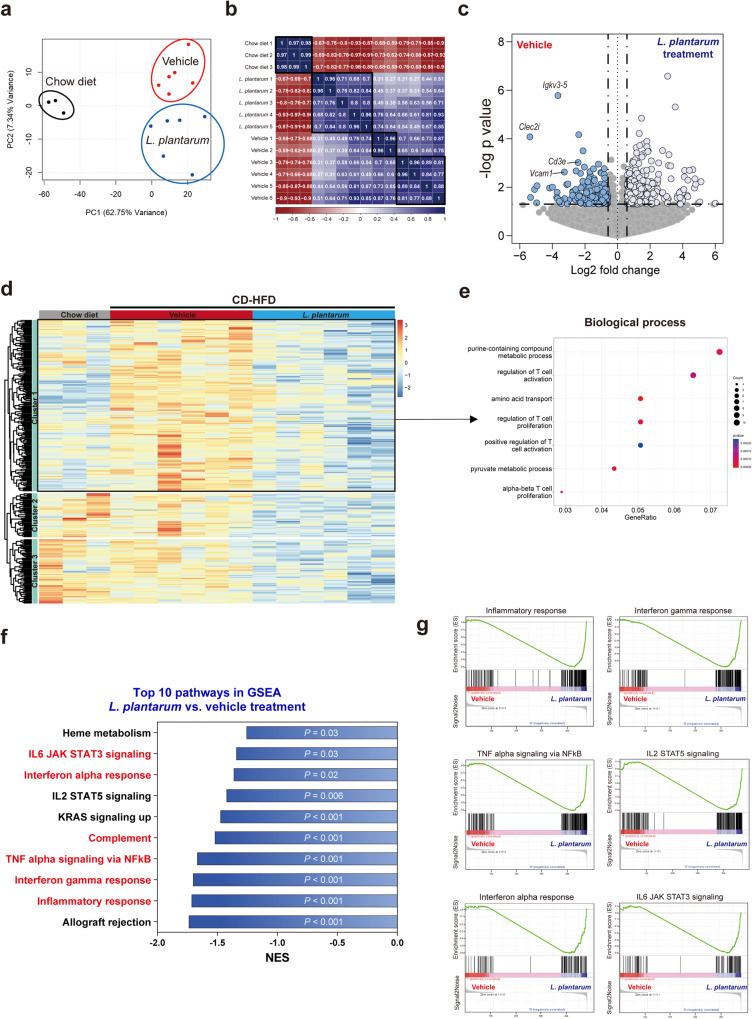
*L. plantarum* treatment resulted in the downregulation of inflammatory genes in the liver. **a** Principal component analysis plot for the RNA-seq data. **b** Pearson correlation coefficients of the RNA-seq data. **c** Volcano plot comparing genes between the *L. plantarum*- and vehicle-treated mice. **d** Heatmap of the differentially expressed genes (DEGs). **e** Gene ontology analysis of DEGs that were downregulated in *L. plantarum*-treated mice (Cluster 1 in **d**). Genes related to inflammation pathways were significantly downregulated by *L. plantarum*. **f** Top 10 GSEA pathways significantly enriched in *L. plantarum*-treated mice compared with vehicle-treated mice. **g** Enrichment plots for inflammation-related pathways in **f**.

We performed RNA-seq analysis to examine the pathways underlying the decreased liver inflammation by *L. plantarum* treatment. We profiled the liver transcriptomes of chow diet-fed, CD-HFD-fed vehicle-treated, and CD-HFD-fed *L. plantarum*-treated mice. Our analysis revealed that CD-HFD caused significant gene expression changes in the liver among the three groups (Fig. 3a, b). We focused on differentially expressed genes (DEGs) between *L. plantarum-* and vehicle-treated mice (Fig. 3c, d). GO analysis of the DEGs indicated that genes related to multiple inflammatory processes, such as regulating T-cell activation and T-cell proliferation, were significantly downregulated upon *L. plantarum* treatment (Fig. 3e). Gene set enrichment analysis also confirmed that multiple inflammatory pathways, namely, the inflammatory response and interferon-gamma response, were deactivated in the livers of *L. plantarum*-treated mice (Fig. 3f, g). To further examine the biological functions of DEGs, we performed a KEGG pathway enrichment analysis, which also revealed inflammatory pathways to be significantly downregulated by *L. plantarum* treatment (Supplementary Fig. 3).

### *L. plantarum* attenuates the inflammatory pathways relevant to human NAFLD

**Fig. 4:**
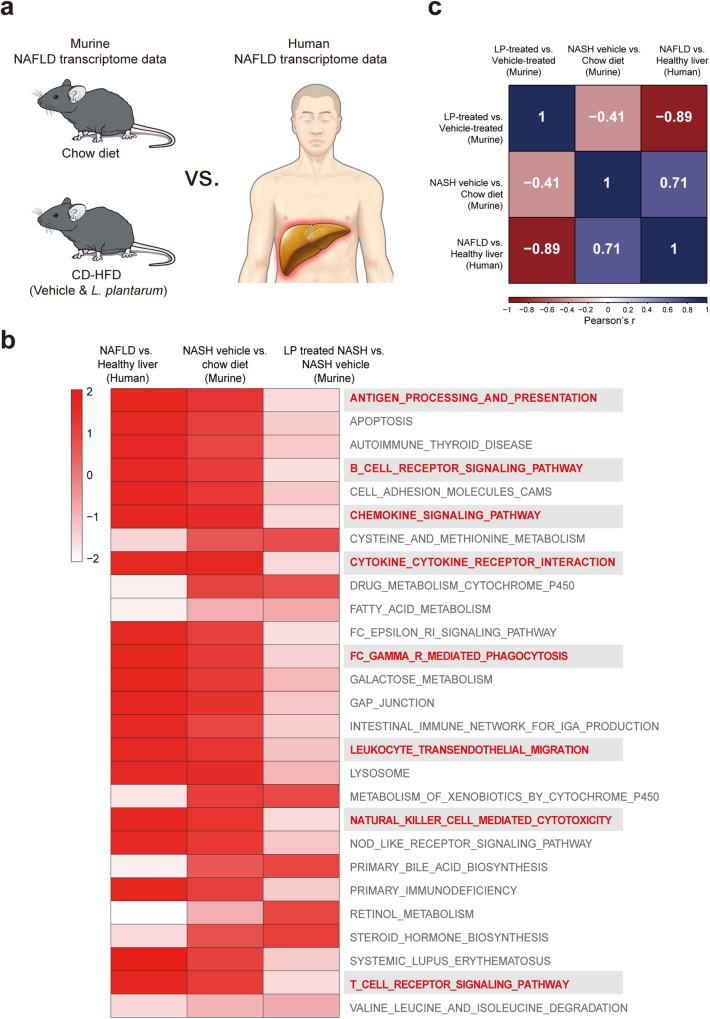
The transcriptome of the murine NAFLD model recapitulates that of human NAFLD. **a** Scheme of comparative transcriptome profiling between murine and human NAFLD. **b** KEGG pathways that are upregulated in human and murine NAFLD. The shade of colors represents the significance of the enrichment score (inflammation-related terms are highlighted in bold red). **c** Pearson correlation coefficients among transcriptomes. NAFLD nonalcoholic fatty liver disease, CD-HFD choline-deficient high-fat diet, LP *Lactobacillus plantarum*.

Increased inflammatory signaling is the hallmark of human NASH^22^. We next tested whether the anti-inflammatory effect of *L. plantarum* was correlated with human NAFLD. Toward achieving this goal, we performed RNA-seq of livers collected from NAFLD patients and compared these data with the murine NASH models used in our study (Fig. 4a). The basic characteristics and phenotypic descriptions are listed in Supplementary Table 3. Several dysregulated inflammatory pathways from KEGG in the NASH murine model were also upregulated in human NAFLD (Fig. 4b, red bold). Moreover, apparent similarity was identified between human “NAFLD vs. healthy liver” and mouse “CD-HFD-fed vehicle group vs. chow diet group” (Fig. 4c, Pearson correlation coefficient = 0.71). These results indicated that the animal model adopted in our study recapitulates human NAFLD. Notably, the commonly upregulated inflammatory pathways in humans and mice were downregulated in the *L. plantarum-*treated mice compared with those in the CD-HFD-fed control mice.

### *L. plantarum* treatment enriched microbial functional genes associated with arginine biosynthesis and elevated serum arginine levels

**Fig. 5:**
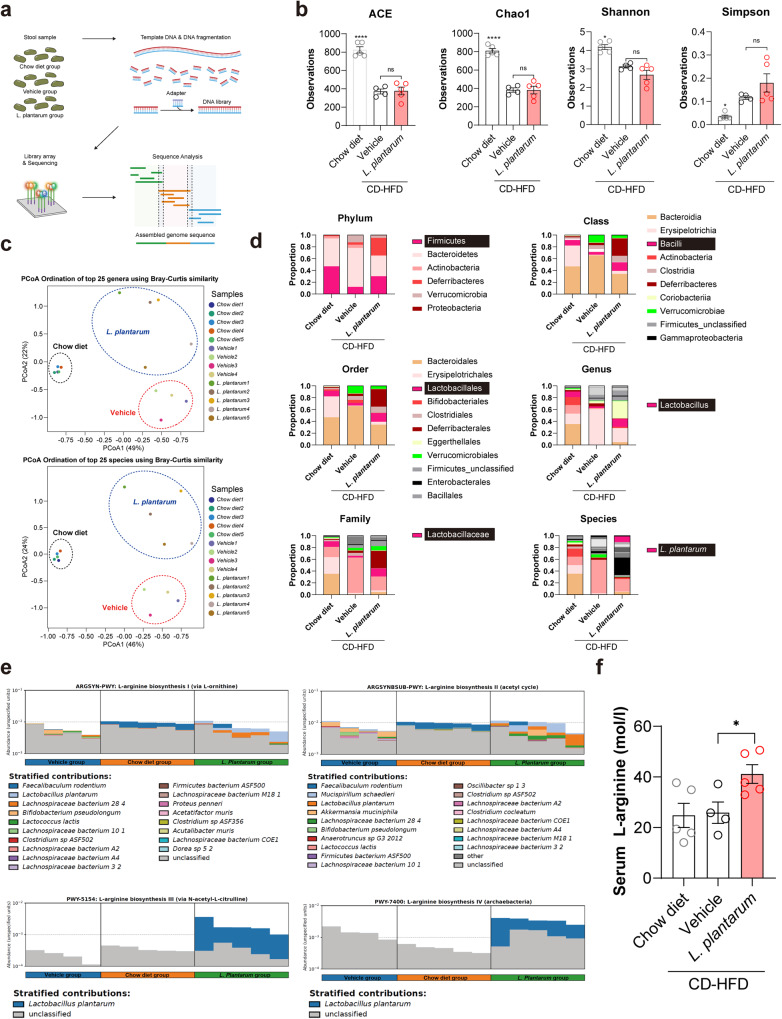
l-Arginine biosynthesis is enhanced by *L. plantarum* treatment in the intestine. **a** Schematic diagram of metagenomic analysis. **b** ACE and Chao1 community richness and Shannon and Simpson community diversity indices. **c** Results of the beta diversity analyses, namely, principal coordinates analysis. **d** Taxonomic compositions. At the species level, *L. plantarum* was identified only in *L. plantarum*-treated mice. **e** The abundance of l-arginine biosynthesis III and IV pathways was enhanced in *L. plantarum*-treated mice. **f** Serum l-arginine levels. *P < 0.05 and ****P < 0.0001 compared with the vehicle-treated mice, using ANOVA and the post hoc Tukey test. CD-HFD choline-deficient high-fat diet.

To identify the underlying mechanism of the *L. plantarum-*mediated attenuation of NASH, we first analyzed the microbial community in the stool to examine whether *L. plantarum* changes the microbial composition (Fig. 5a). The ACE and Chao1 community richness indices of the gut microbiome in CD-HFD-fed vehicle- and *L. plantarum-*treated mice were decreased compared with that in chow diet-fed mice. The same pattern was also observed in the Shannon and Simpson diversity indices (Fig. 5b). These results corroborated the findings of previous studies suggesting that the richness and diversity of the microbiomes in NAFLD patients were lower than those in healthy individuals^23,24^. However, *L. plantarum* treatment failed to recover the decreased richness and diversity. Beta diversity analysis of the relationship among microbiome taxonomic profiles confirmed that the bacterial community structures at both the genus and species levels differed by food and *L. plantarum* treatment (Fig. 5c). We then investigated the microbial composition to detect *L. plantarum* only in the *L. plantarum*-treated mice (Fig. 5d and Supplementary Table 4). The highlighted text indicates each level to which *L. plantarum* belongs (Fig. 5d). Moreover, the metagenomic data were compared using the UniRef90 gene family (Supplementary Fig. 4) and KEGG database to investigate the functional distribution in the mouse gut microbiome with respect to the genomic contents and functional activity. l-Arginine biosynthesis was the most significantly abundant function in *L. plantarum-*treated mice compared with vehicle-treated mice. Four pathways are associated with l-arginine biosynthesis: (1) l-arginine biosynthesis I via l-ornithine, (2) l-arginine biosynthesis II via the acetyl cycle, (3) l-arginine biosynthesis III via *N*-acetyl-l-citrulline, and (4) l-arginine biosynthesis IV via archaebacteria. Among these pathways, l-arginine biosynthesis III via *N*-acetyl-l-citrulline and l-arginine biosynthesis IV via archaebacteria were significantly more enriched upon *L. plantarum* treatment (Fig. 5e). We also measured serum l-arginine levels using an ELISA kit to investigate whether *L. plantarum* affects l-arginine levels; the results revealed that serum l-arginine concentrations were significantly higher in *L. plantarum-*treated mice than in other mice (Fig. 5f).

### *L. plantarum* treatment intensifies intestinal barrier function

Next, we investigated whether the intestinal barrier protective function of *L. plantarum*^25^ contributes to its therapeutic effect on NASH. To evaluate intestinal epithelial integrity, we measured the fluorescence intensity of FD4K, the diffusion of which indicates paracellular permeability, in the serum of chow diet-fed and CD-HFD-fed vehicle- and *L. plantarum*-treated mice. The FD4K fluorescence was increased by CD-HFD intake but was returned to the control level by *L. plantarum* treatment (Supplementary Fig. 5a). Elevated serum endotoxin levels are often present in NAFLD patients who have disrupted intestinal barrier function^26^. Therefore, we next examined the endotoxin levels, which were elevated in the CD-HFD group but reduced upon *L. plantarum* treatment (Supplementary Fig. 5b). To confirm the effect of *L. plantarum* on intestinal permeability, we performed RNA-seq analysis using ileal tissues of mice (Supplementary Fig. 5c). The transcriptomes of different treatments were significantly different (Supplementary Fig. 5d, e), with genes related to intestinal barrier function (GO:0090557) among the DEGs being significantly decreased in CD-HFD-fed mice compared with chow diet-fed mice (Supplementary Fig. 5f). The heatmap confirmed that these genes were downregulated by CD-HFD but rescued to the control level by *L. plantarum* (Supplementary Fig. 5f), as depicted in the volcano plot (Supplementary Fig. 5g).

To further confirm the effect of *L. plantarum* on intestinal permeability in vitro, we pretreated Caco-2 enterocytes with LPS, causing intestinal barrier disruption^27^, for 2 h before *L. plantarum* treatment. We measured the expression of tight junction (TJ) proteins, such as E-cadherin and occludin, using immunoblotting to determine how TJ proteins were affected by *L. plantarum* treatment. Concordantly, challenge with LPS significantly decreased the abundance of E-cadherin and Occludin in Caco-2 cells. However, treatment with *L. plantarum* significantly restored their abundance (Supplementary Fig. 6a, b). Collectively, our results demonstrated that the defective intestinal barrier resulting from CD-HFD intake could be rescued by *L. plantarum* treatment.

### The preventive effect of *L. plantarum* in the NAFLD mouse model

We also investigated the preventive effect of *L. plantarum* on early-phase NAFLD in the NAFL mouse model. Unlike the NASH models used in earlier studies, we administered *L. plantarum* for eight weeks after eight weeks of CD-HFD feeding. Similar to the results in the NASH murine model (Fig. 1a), the administration of *L. plantarum* did not affect the body weights of the NAFL model mice (Supplementary Fig. 7a). Histological analysis revealed that *L. plantarum* inhibited fat accumulation compared with that of the vehicle group (Supplementary Fig. 7b). In addition, serum biochemical tests suggested that the levels of the liver injury markers AST and ALT were lower in *L. plantarum*-treated mice (Supplementary Fig. 7c). We also performed the IPITT and OGTT to evaluate the preventive effects of *L. plantarum* on glucose homeostasis. Concordantly, treatment with *L. plantarum* reduced insulin resistance compared with that of the vehicle treatment in the NAFL model (Supplementary Fig. 7d, e).

### l-Arginine is sufficient to alleviate NASH

**Fig. 6:**
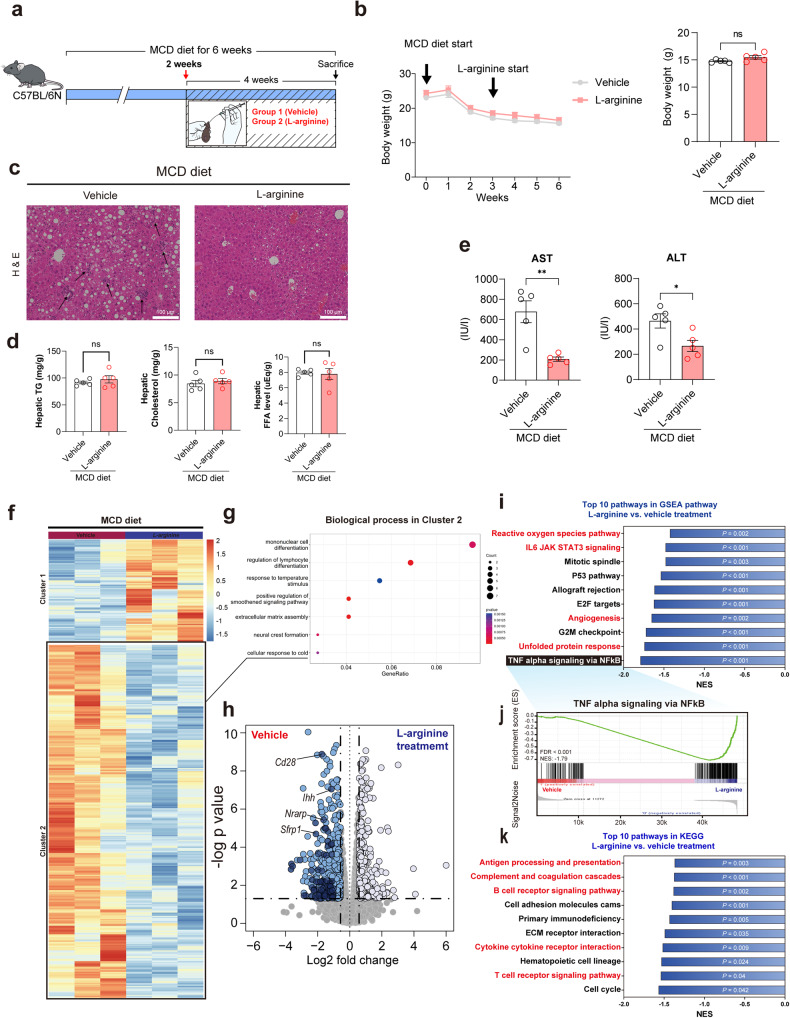
l-Arginine treatment ameliorates liver inflammation in MCD-induced NASH mice. **a** Experimental scheme. **b** Changes in body weight over time. **c** Representative photomicrographs of liver sections stained with H&E. Scale bar, 100 μm. **d** Hepatic lipid levels. **e** Serum biochemistry levels. **f** Heatmap of differentially expressed genes (DEGs) from RNA-seq analysis. **g** Gene ontology analysis of DEGs that were downregulated in l-arginine-treated mice. **h** Volcano plot depicting genes in **g**. Inflammatory genes are marked in deep blue. **i**–**k** Top 10 GSEA pathways enriched in l-arginine-treated mice compared with vehicle-treated mice based on the hallmark (**i**) and KEGG (**k**) pathways. GSEA enrichment plot for TNF-alpha signaling via the nuclear transcription factor-kappa B (NF-κB) pathway (**j**). *P < 0.05 and **P < 0.01 compared with the vehicle-treated mice, using ANOVA and the post hoc Tukey test. MCD methionine-choline deficient.

Significant induction of the gene encoding l-arginine biosynthesis and increased l-arginine levels were observed in *L. plantarum-*treated mice, leading us to hypothesize that l-arginine mediates the effect of *L. plantarum* via the gut-liver axis. To evaluate this hypothesis, we examined the effect of l-arginine alone on mice and liver organoids. First, we tested the therapeutic effect of l-arginine in NASH mouse models, where mice were fed a methionine-choline-deficient (MCD) diet for two weeks and were fed l-arginine daily by oral gavage for four weeks (Fig. 6a). We observed that the body weights of l-arginine-treated mice were not different from those of vehicle-treated mice (Fig. 6b). Histological examination by H&E staining revealed that l-arginine decreased liver inflammation, as demonstrated by reduced neutrophil infiltration (Fig. 6c), whereas there were no significant differences in the hepatic lipid content between l-arginine-treated and vehicle-treated mice (Fig. 6d). In addition, the serum levels of ASL and ALT were lower in the l-arginine-treated mice than in the vehicle-treated mice (Fig. 6e). To explore the underlying mechanisms associated with l-arginine treatment, we compared the liver transcriptomes between MCD diet-fed l-arginine-treated and vehicle-treated mice. DEG and GO analysis revealed that several inflammatory genes and pathways were downregulated by l-arginine treatments (Cluster 2 genes in 6 f and blue dots in 6 h) (Fig. 6f–h). Compared with those in the vehicle-treated mice, the top 10 most significantly downregulated gene sets in the l-arginine–treated mice are displayed according to reference gene sets (Fig. 6i, k and Supplementary Fig. 8). Interestingly, we found that TNF-alpha signaling via the nuclear transcription factor-kappa B (NF-κB) pathway was most significantly downregulated with the l-arginine treatment (Fig. 6i, j), which is consistent with the previous result of downregulation of the same gene set by *L. plantarum* treatment (Fig. 3f).

**Fig. 7:**
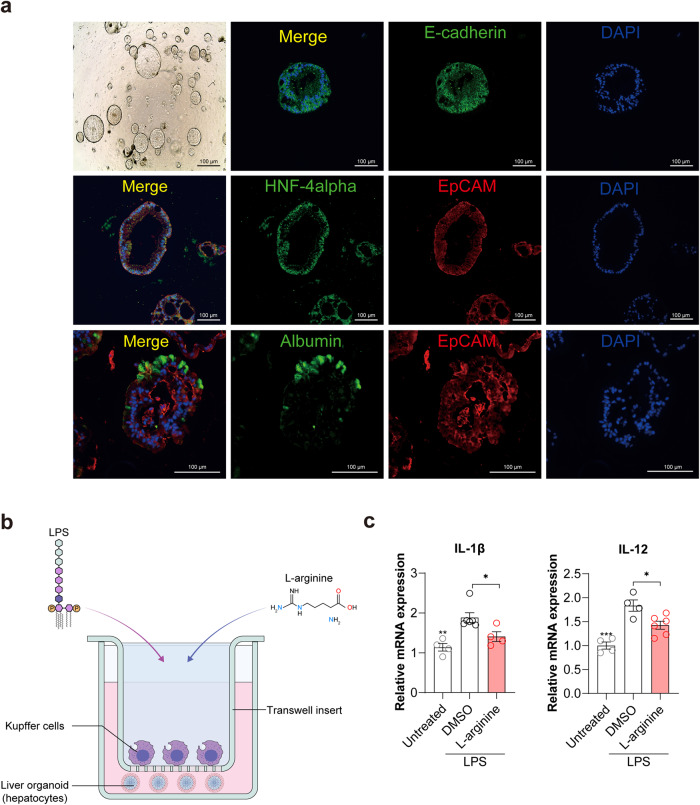
l-Arginine reduces LPS-induced inflammation in liver organoids. **a** Representative photomicrographs and immunofluorescence images for liver markers in the murine liver organoid. Scale bars = 100 μm. **b** Schematic diagram of the coculturing experiment. **c** Expression of inflammatory marker genes in the DMSO- and l-arginine-treated liver organoids. *P < 0.05 compared with the DMSO-treated group, using ANOVA and the post hoc Tukey test. IL-1β, interleukin 1 beta; IL-12, interleukin 12.

We also examined the effect of l-arginine on liver inflammation in vitro by coculturing Kupffer cells and liver organoids in a Transwell system to mimic the NASH microenvironment (Fig. 7a, b). Immunofluorescence assays revealed that the liver organoids were positive for several liver markers (E-cadherin, HNF4-alpha, and albumin) (Fig. 7a). The organoids and cells were then exposed to lipopolysaccharide (LPS) for 24 h and were treated with l-arginine or DMSO for 24 h (Fig. 7b). l-Arginine-treated organoids exhibited lower IL-1β and IL-12 mRNA levels than those of vehicle-treated control organoids (Fig. 7c). These results collectively indicated that l-arginine could be a potential mediator of the therapeutic effect of *L. plantarum* and sufficient to alleviate NASH. To further assess the role of l-arginine in human NAFLD, we examined whether l-arginine levels changed with NAFLD progression. Blood samples were collected from a total of 19 individuals, including 14 NAFLD patients (7 with simple steatosis and 7 with NASH and fibrosis) and 5 healthy living donors. Intriguingly, as NAFLD progressed, l-arginine levels declined (Supplementary Fig. 9). Despite the fact that these results do not establish a causal relationship between l-arginine and the development of NAFLD, they suggest that l-arginine may be associated with NAFLD and may play a significant role in its progression, even when considering human data.

## Discussion

In the present study, we highlighted the therapeutic potential of *L. plantarum* in the CD-HFD-induced NASH murine model. We also demonstrated that this effect of *L. plantarum* is mediated by L-arginine. To the best of our knowledge, we elucidated the underlying mechanism of *L. plantarum* treatment based on the gut-liver axis for the first time.

Various mouse models have been used to study NAFLD, with each displaying one or more features of human NAFLD and its consequences, such as fibrosis, cirrhosis, and the development of HCC^28^. However, appropriate mouse models for studying NASH development have not been established. The frequent choline deficiency in NASH patients^29^ led us to use a CD-HFD-induced NASH mouse model that manifests the whole spectrum of NAFLD and recapitulates critical features of NAFLD in humans. Moreover, the transcriptomic profiles between human NAFLD patients and our murine mouse model were similar, as evidenced from the RNA-seq analysis (Fig. 4).

**Fig. 8:**
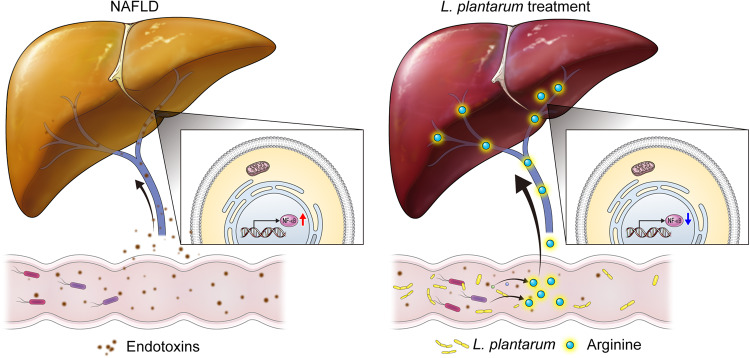
Scheme showing that *L. plantarum* treatment improved liver inflammation concurrently by modulating the NF-kB pathway.

Although *L. plantarum* reportedly has beneficial effects in several NAFLD animal models^30,31^, the underlying mechanisms are not well understood. In this study, we demonstrated that *L. plantarum* administration ameliorates liver inflammation in a NASH murine model. Oral administration of *L. plantarum* significantly improved NASH-related metrics, namely, liver histology results, NAFLD activity scores, and liver inflammation. The liver histology analysis identified decreased liver inflammation, decreased serum liver enzyme levels, and the downregulation of inflammation-related genes, as confirmed by liver transcriptome analysis. Counterintuitively, we also showed that l-arginine mediates this effect via the gut-liver axis. By profiling the functions of microbial communities using whole metagenomic sequencing, we observed that l-arginine biosynthesis pathways III and IV were more enriched in *L. plantarum*-treated mice than in chow-diet-fed or CD-HFD-fed vehicle-treated mice. We confirmed the increased serum l-arginine levels in *L. plantarum*-treated mice.

l-Arginine is a basic semiessential amino acid that exerts several roles, such as blood flow regulation and host defense^32,33^. The known underlying mechanisms for these effects of l-arginine involve multiple nitric oxide-dependent pathways that support the whole-body oxidation of fatty acids and glucose^34^. Although l-arginine is typically synthesized via the intestinal-renal axis^35^, l-arginine is also biosynthesized in the intestine and by lactic acid bacteria, such as *L. plantarum*^36^. *L. plantarum* harbors l-arginine biosynthesis-related genes^37^. Metagenomic analysis during *L. plantarum* treatment confirmed its presence only in the intestine of *L. plantarum*-treated mice. This result illustrates that *L. plantarum*, present in the intestinal microbiome, can be a source of l-arginine. Recent evidence suggests that l-arginine is essential in various biological processes, such as immune responses^38–43^. Furthermore, several studies have reported that l-arginine exerts a protective effect in many liver injury models^44,45^. Thus, we hypothesized that l-arginine might potentially mediate the therapeutic effect of *L. plantarum* on NASH. To test this hypothesis, we orally administered l-arginine to MCD diet-fed mice, another dietary model of NASH. Histological and serum biochemical analyses revealed that l-arginine reduced liver inflammation in MCD diet-induced NASH mice. Moreover, we noticed that the NF-κB pathway was downregulated not only by l-arginine treatment (Fig. 6i) but also by *L. plantarum* treatment (Fig. 3f). Several studies previously showed that l-arginine attenuated NF-kB pathway activation by modulating nitric oxide metabolism^46,47^. These data support the idea that the anti-inflammatory effect of *L. plantarum* is mediated by l-arginine (Fig. 8).

Collectively, these results support the hypothesis that the therapeutic effect of *L. plantarum* on NASH is mediated by the l-arginine that *L. plantarum* produces. Moreover, we noticed that the arginine biosynthesis pathway was decreased in the liver transcriptome of the *L. plantarum*-treated mice compared with vehicle-treated mice (Supplementary Fig. 10). One possible explanation for this result is that l-arginine production in the liver in *L. plantarum*-treated mice would be decreased by exposure to an exogenous source of l-arginine, such as the intestine.

The strengths of this study that we wish to highlight are that we present a potential role of *L. plantarum* in improving NAFLD, which was mediated by l-arginine, and add to the established underlying mechanisms of *L. plantarum*, such as intensifying intestinal barrier function and reducing systemic LPS^48,49^. Although we demonstrated that both *L. plantarum* and l-arginine improved liver inflammation, *L. plantarum* would be a more promising agent considering that half of dietary l-arginine is degraded by the small intestine during first-pass metabolism^50^, in addition to the additional beneficial effect of *L. plantarum*, which refers to improving the intestinal barrier function (Supplementary Figs. 5–6). Considering that previous studies have demonstrated that gut microbiota-derived endotoxins may play a role in the progression of NAFLD from simple fat deposition to steatohepatitis^51–54^, *L. plantarum* may confer benefits not only by increasing the l-arginine content but also by modulating intestinal barrier permeability, thereby decreasing serum endotoxin levels. Both effects can contribute to attenuating NASH.

However, our findings are limited because while the CD-HFD-induced NASH murine model recapitulates human NAFLD, several differences exist between the murine model and humans, including differences in the gut mycobiome populations. The increased l-arginine production with the administration of *L. plantarum* observed in this study should be confirmed in humans. The effect of l-arginine in alleviating human NASH symptoms also needs evaluation. Therefore, it would be difficult to translate our findings directly into therapeutic agents for human NAFLD. Nevertheless, based on our results, we believe that *L. plantarum* could be considered a potential therapeutic strategy against NASH.

Collectively, our results demonstrated that *L. plantarum* treatment enhances l-arginine synthesis in the intestine, increasing systemic circulating l-arginine levels and thereby ameliorating NASH-related phenotypes. *L. plantarum* colonizes the gut microbiome and contributes to l-arginine biosynthesis. We also confirmed that l-arginine adequately reduces liver inflammation, indicating its role in mediating the therapeutic effect of *L. plantarum*. However, further studies should be conducted to validate these findings in NAFLD patients.

### Supplementary information

Supplementary Information

## Data Availability

The RNA-seq datasets generated in this study are available from the Korean Nucleotide Archive (KoNA, https://kobic.re.kr/kona) (accession ID: PRJKA220516). The datasets generated during and/or analyzed during the current study are available from the corresponding author on reasonable request.

## References

[1] Younossi Z (2018). Global burden of NAFLD and NASH: trends, predictions, risk factors and prevention. Nat. Rev. Gastroenterol. Hepatol..

[2] Arab JP, Arrese M, Trauner M (2018). Recent insights into the pathogenesis of nonalcoholic fatty liver disease. Annu. Rev. Pathol..

[3] Chalasani N (2012). The diagnosis and management of non‐alcoholic fatty liver disease: Practice Guideline by the American Association for the Study of Liver Diseases, American College of Gastroenterology, and the American Gastroenterological Association. Hepatology.

[4] Lindenmeyer CC, McCullough AJ (2018). The natural history of nonalcoholic fatty liver disease—an evolving view. Clin. Liver Dis..

[5] Rinella ME, Sanyal AJ (2016). Management of NAFLD: a stage-based approach. Nat. Rev. Gastroenterol. Hepatol..

[6] European Association for the Study of the Liver (EASL),* European Association for the Study of Diabetes (EASD), & European Association for the Study of Obesity (EASO) EASL-EASD-EASO Clinical Practice Guidelines for the management of non-alcoholic fatty liver disease. *Obes. Facts* **9**, 65–90 (2016). 10.1159/000443344 PMC5644799 27055256

[7] Kang SH (2021). KASL clinical practice guidelines: Management of nonalcoholic fatty liver disease. Clin. Mol. Hepatol..

[8] Leung C, Rivera L, Furness JB, Angus PW (2016). The role of the gut microbiota in NAFLD. Nat. Rev. Gastroenterol. Hepatol..

[9] Suez J, Zmora N, Segal E, Elinav E (2019). The pros, cons, and many unknowns of probiotics. Nat. Med..

[10] Cohen LJ (2017). Commensal bacteria make GPCR ligands that mimic human signalling molecules. Nature.

[11] Marra F, Svegliati-Baroni G (2018). Lipotoxicity and the gut-liver axis in NASH pathogenesis. J. Hepatol..

[12] Parada Venegas D (2019). Short chain fatty acids (SCFAs)-mediated gut epithelial and immune regulation and its relevance for inflammatory bowel diseases. Front. Immunol..

[13] Qiu L, Tao X, Xiong H, Yu J, Wei H (2018). Lactobacillus plantarum ZDY04 exhibits a strain-specific property of lowering TMAO via the modulation of gut microbiota in mice. Food Funct..

[14] Barnett AM, Roy NC, Cookson AL, McNabb WC (2018). Metabolism of caprine milk carbohydrates by probiotic bacteria and Caco-2: HT29–MTX epithelial co-cultures and their impact on intestinal barrier integrity. Nutrients.

[15] Behera, S. S., Ray, R. C. & Zdolec, N. Lactobacillus plantarum with functional properties: an approach to increase safety and shelf-life of fermented foods. *Biomed. Res. Int*. **2018**, 9361614 (2018). 10.1155/2018/9361614 PMC5994577 29998137

[16] Zhao Z (2019). Lactobacillus plantarum NA136 improves the non-alcoholic fatty liver disease by modulating the AMPK/Nrf2 pathway. Appl. Microbiol. Biotechnol..

[17] Lee NY (2021). Lactobacillus attenuates progression of nonalcoholic fatty liver disease by lowering cholesterol and steatosis. Clin. Mol. Hepatol..

[18] Love MI, Huber W, Anders S (2014). Moderated estimation of fold change and dispersion for RNA-seq data with DESeq2. Genome Biol..

[19] Wieland A, Frank D, Harnke B, Bambha K (2015). Systematic review: microbial dysbiosis and nonalcoholic fatty liver disease. Aliment. Pharmacol. Ther..

[20] Lattouf R (2014). Picrosirius red staining: a useful tool to appraise collagen networks in normal and pathological tissues. J. Histochem. Cytochem..

[21] Ayala JE (2010). Standard operating procedures for describing and performing metabolic tests of glucose homeostasis in mice. Dis. Model. Mech..

[22] Cheung O (2008). Nonalcoholic steatohepatitis is associated with altered hepatic MicroRNA expression. Hepatology.

[23] Betrapally NS, Gillevet PM, Bajaj JS (2016). Changes in the intestinal microbiome and alcoholic and nonalcoholic liver diseases: causes or effects?. Gastroenterology.

[24] Zhu L (2013). Characterization of gut microbiomes in nonalcoholic steatohepatitis (NASH) patients: a connection between endogenous alcohol and NASH. Hepatology.

[25] Jones RM (2015). Lactobacilli modulate epithelial cytoprotection through the Nrf2 pathway. Cell Rep..

[26] Harte AL (2010). Elevated endotoxin levels in non-alcoholic fatty liver disease. J. Inflamm..

[27] Zhang D, Wen J, Zhou J, Cai W, Qian L (2019). Milk fat globule membrane ameliorates necrotizing enterocolitis in neonatal rats and suppresses lipopolysaccharide‐induced inflammatory response in IEC‐6 enterocytes. JPEN J. Parenter. Enter. Nutr..

[28] Friedman SL, Neuschwander-Tetri BA, Rinella M, Sanyal AJ (2018). Mechanisms of NAFLD development and therapeutic strategies. Nat. Med..

[29] Corbin KD, Zeisel SH (2012). Choline metabolism provides novel insights into non-alcoholic fatty liver disease and its progression. Curr. Opin. Gastroenterol..

[30] Zhao Z (2020). Lactobacillus plantarum NA136 ameliorates nonalcoholic fatty liver disease by modulating gut microbiota, improving intestinal barrier integrity, and attenuating inflammation. Appl. Microbiol. Biotechnol..

[31] Park E-J (2020). Beneficial effects of Lactobacillus plantarum strains on non-alcoholic fatty liver disease in high fat/high fructose diet-fed rats. Nutrients.

[32] Gogoi M, Datey A, Wilson KT, Chakravortty D (2016). Dual role of arginine metabolism in establishing pathogenesis. Curr. Opin. Microbiol..

[33] Hou Y, Wu G (2017). Nutritionally nonessential amino acids: a misnomer in nutritional sciences. Adv. Nutr..

[34] Jobgen WS, Fried SK, Fu WJ, Meininger CJ, Wu G (2006). Regulatory role for the arginine–nitric oxide pathway in metabolism of energy substrates. J. Nutr. Biochem..

[35] Wu G, Morris SM (1998). Arginine metabolism: nitric oxide and beyond. Biochem. J..

[36] Bringel F, Frey L, Boivin S, Hubert JC (1997). Arginine biosynthesis and regulation in Lactobacillus plantarum: the carA gene and the argCJBDF cluster are divergently transcribed. J. Bacteriol..

[37] Nicoloff H, Arsene-Ploetze F, Malandain C, Kleerebezem M, Bringel F (2004). Two arginine repressors regulate arginine biosynthesis in Lactobacillus plantarum. J. Bacteriol..

[38] Han J (2009). Dietary L-arginine supplementation alleviates immunosuppression induced by cyclophosphamide in weaned pigs. Amino Acids.

[39] Wu, T. et al. Arginine relieves the inflammatory response and enhances the casein expression in bovine mammary epithelial cells induced by lipopolysaccharide. *Mediat. Inflamm*. **2016**, 9618795 (2016). 10.1155/2016/9618795 PMC4821974 27110069

[40] Hnia K (2008). L-arginine decreases inflammation and modulates the nuclear factor-κB/matrix metalloproteinase cascade in mdx muscle fibers. Am. J. Pathol..

[41] Lan J (2020). L-arginine ameliorates lipopolysaccharide-induced intestinal inflammation through inhibiting the TLR4/NF-κB and MAPK pathways and stimulating β-defensin expression in vivo and in vitro. J. Agric. Food Chem..

[42] de Jonge WJ (2002). Arginine deficiency affects early B cell maturation and lymphoid organ development in transgenic mice. J. Clin. Invest..

[43] Li P, Yin Y-L, Li D, Kim SW, Wu G (2007). Amino acids and immune function. Br. J. Nutr..

[44] Giovanardi RO, Rhoden EL, Cerski CT, Salvador M, Kalil AN (2009). Pharmacological preconditioning using intraportal infusion of L-arginine protects against hepatic ischemia reperfusion injury. J. Surg. Res..

[45] Muriel P, González P (1998). Liver damage induced by acute cholestasis in the rat is ameliorated partially by L-arginine. Comp. Biochem. Physiol. Part C: Pharmacol. Toxicol. Endocrinol..

[46] Meng Q, Cooney M, Yepuri N, Cooney RN (2017). L-arginine attenuates Interleukin-1β (IL-1β) induced Nuclear Factor Kappa-Beta (NF-κB) activation in Caco-2 cells. PLoS ONE.

[47] Visigalli R (2004). The stimulation of arginine transport by TNFα in human endothelial cells depends on NF-κB activation. Biochim. Biophy. Acta (BBA)-Biomembr..

[48] Liu, Z. et al. Lactobacillus plantarum 23-1 improves intestinal inflammation and barrier function through the TLR4/NF-κB signaling pathway in obese mice. *Food Funct*. **13**, 5971-5986 (2022). 10.1039/d1fo04316a 35546499

[49] Wang J (2018). Probiotic Lactobacillus plantarum promotes intestinal barrier function by strengthening the epithelium and modulating gut microbiota. Front. Microbiol..

[50] Wu G (2007). Pharmacokinetics and safety of arginine supplementation in animals. J. Nutr..

[51] Tsukumo DM (2007). Loss-of-function mutation in Toll-like receptor 4 prevents diet-induced obesity and insulin resistance. Diabetes.

[52] Miura K (2010). Toll-like receptor 9 promotes steatohepatitis by induction of interleukin-1β in mice. Gastroenterology.

[53] Henao-Mejia J (2012). Inflammasome-mediated dysbiosis regulates progression of NAFLD and obesity. Nature.

[54] Männistö V (2019). Serum lipopolysaccharides predict advanced liver disease in the general population. JHEP Rep..

