# Supplementary material for: Lactobacillus plantarum ameliorates NASH-related inflammation by upregulating l-arginine production
Source: Exp Mol Med. 2023 Nov 1;55(11):2332–45. doi: 10.1038/s12276-023-01102-0 (PMC10689779; doi:10.1038/s12276-023-01102-0)
Supplement: Supplementary file 1 — Supplementary Information [file 12276_2023_1102_MOESM1_ESM.pdf]

SUPPLEMENTARY INFORMATION

Lactobacillus plantarum ameliorates NASH-related inflammation by upregulating L-arginine production

Dong Yun Kim[1,2,3], Jun Yong Park[2,3], and Heon Yung Gee[1,4,5]

[1] Department of Pharmacology, Yonsei University College of Medicine, Seoul, Republic of South Korea

[2] Division of Gastroenterology, Department of Internal Medicine, Yonsei University College of Medicine, Seoul, Republic of South Korea

[3] Yonsei Liver Center, Severance Hospital, Seoul, Republic of South Korea

[4] Graduate School of Medical Science, Brain Korea 21 Project, Yonsei University College of Medicine, Seoul, Republic of South Korea

[5] Woo Choo Lee Institute for Precision Drug Development

SUPPLEMENTARY FIGURES

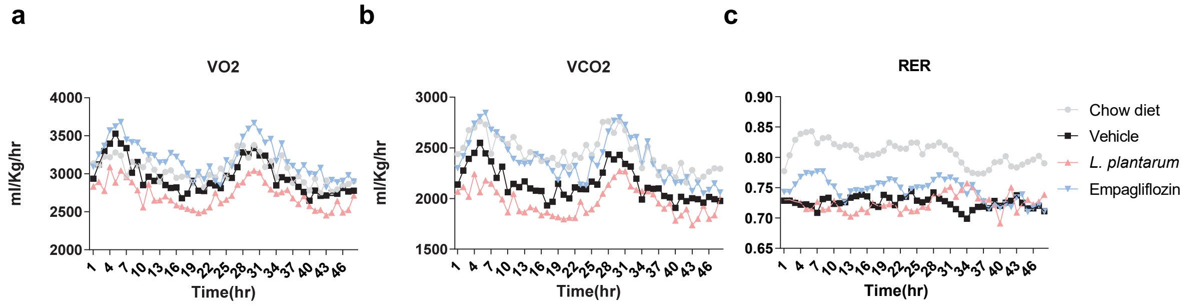

Supplementary Fig. 1. Energy homeostasis during treatment.

(a) Oxygen consumption rate (VO2), (b) carbon dioxide release rate (VCO2), (c) respiratory exchange ratio measured using a metabolic cage after treatment (n = 3–4 per group).

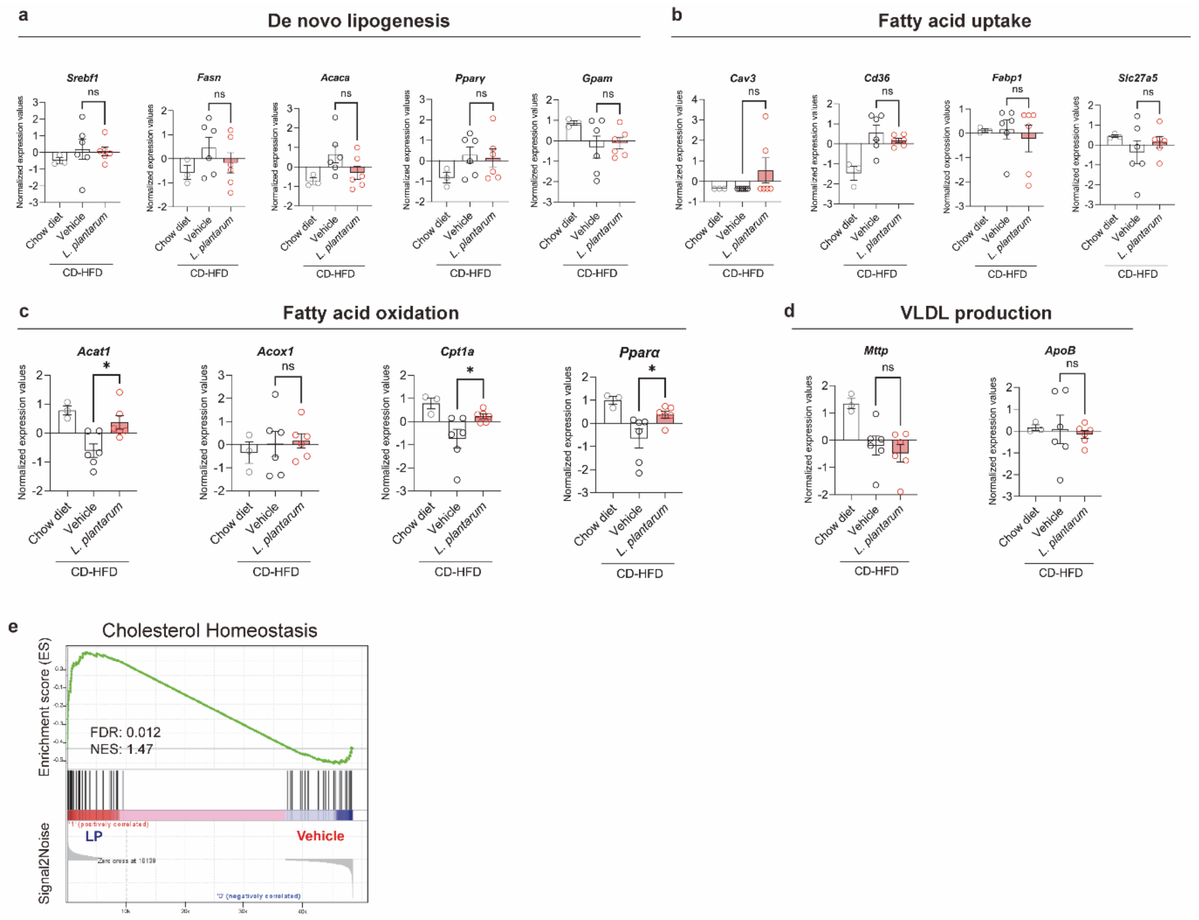

Supplementary Fig. 2. ***L. plantarum*** **treatment regulates hepatic lipid metabolism.**

(a-d) mRNA levels of genes implicated in lipid metabolism in the liver, as measured by real-time (RT)-PCR; de novo lipogenesis (a), fatty acid uptake (b), fatty acid oxidation (c) and VLDL generation (d). (e) Enrichment plot for lipid metabolism of the transcriptome data in the *L. plantarum* group relative to the vehicle group. Data are expressed as mean ± SD. *$P < 0.05$ compared to the vehicle-treated group, using the post-hoc pairwise Mann-Whitney U tests after Kruskal-Wallis test with Bonferroni correction. VLDL, very low-density lipoprotein.

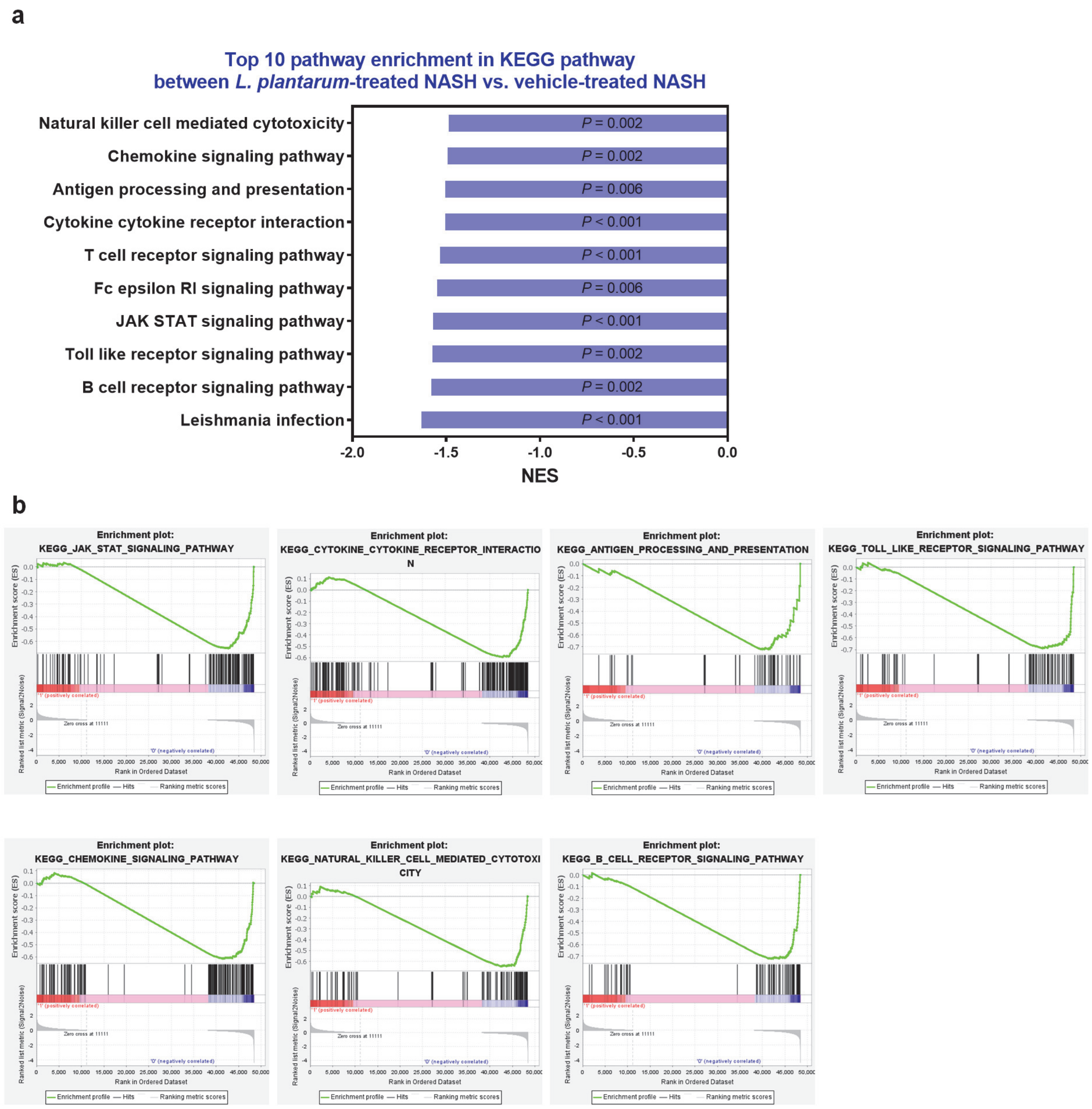

Supplementary Fig. 3. KEGG pathway enrichment analysis results of the transcriptome data in the *L. plantarum* treatment group.

(a) Top 10 enriched pathways of the transcriptome data in the *L. plantarum* treatment group compared with the vehicle group. (b) Enrichment plot for inflammation-related seven terms.

a

DNA Paired end reads

	Raw	Trim	mouse_C57BL_6NJ
Chow diet1	42,701,488	33,089,770	32,277,464
Chow diet2	38,434,691	29,547,908	28,557,021
Chow diet3	42,889,238	32,832,987	32,396,373
Chow diet4	40,790,788	31,442,747	29,735,225
Chow diet5	36,420,531	27,993,824	27,025,363
Vehicle1	38,892,088	29,331,966	19,174,193
Vehicle2	39,867,383	30,505,097	10,712,920
Vehicle3	41,975,933	32,997,000	29,012,758
Vehicle4	41,994,103	37,131,260	33,303,885
LP1	41,234,664	36,518,788	28,312,811
LP2	38,858,437	34,286,349	31,014,413
LP3	39,081,698	34,826,003	6,567,359
LP4	39,753,680	34,619,213	20,355,131
LP5	42,455,157	37,712,656	2,070,638

b

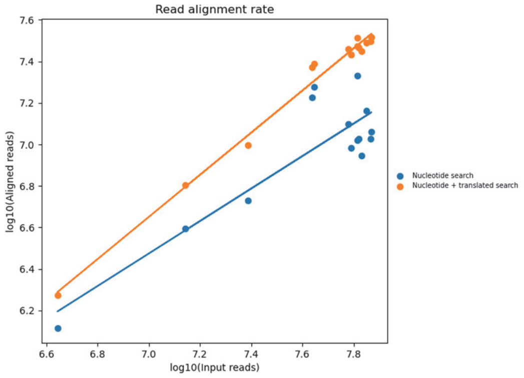

c

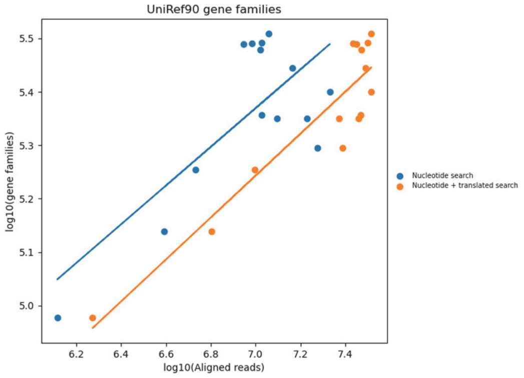

Supplementary Fig. 4. Quality control for metagenomic shotgun analysis.

(a) The number of reads filtered at each QC step. Summary read values of metagenomic data after filtering. (b) Map reads to alignment rate. (c) Align reads to a UniRef90 database. Abbreviation: LP, *Lactobacillus plantarum*.

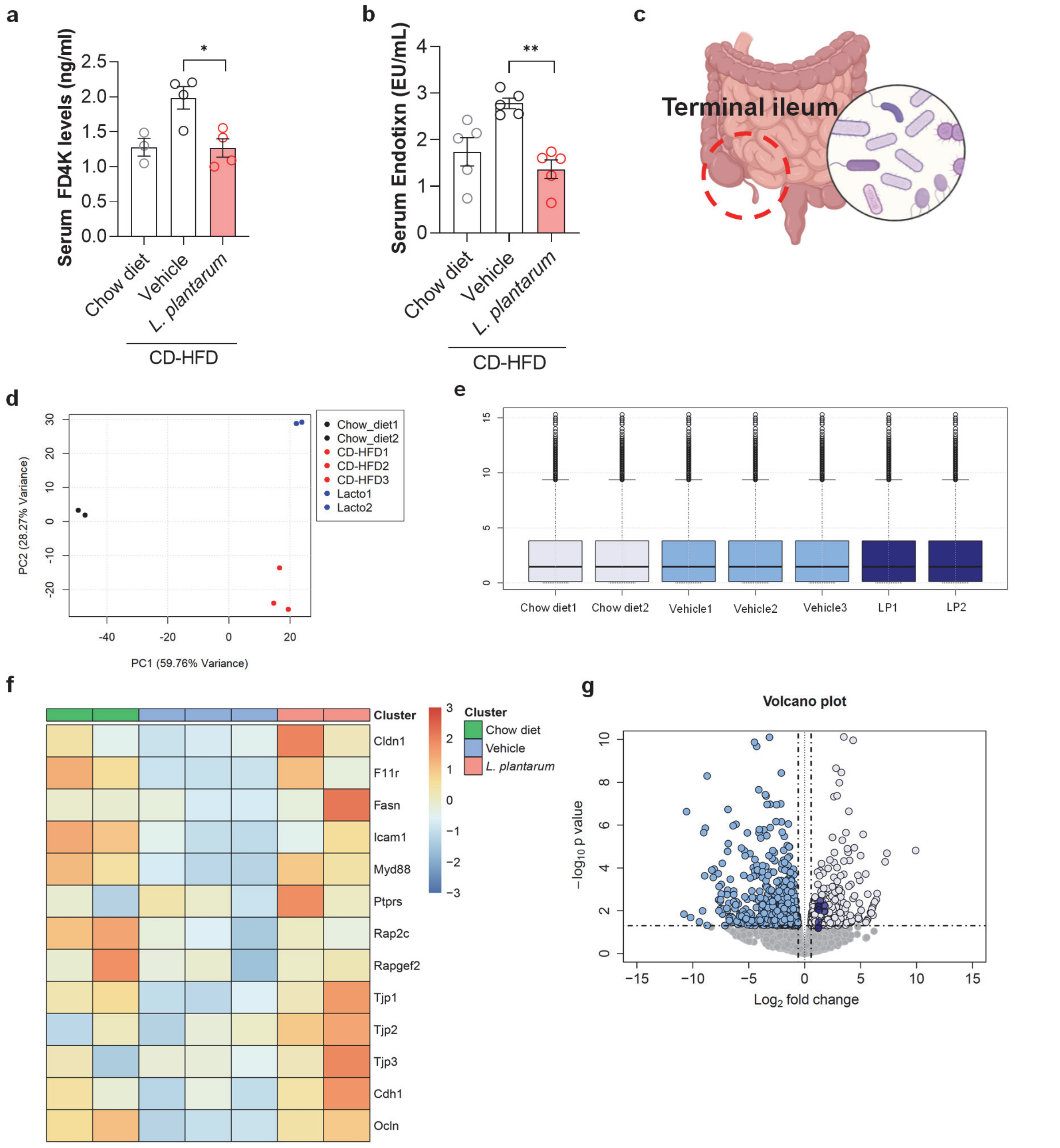

Supplementary Fig. 5. Improved intestinal permeability and transcriptomic changes of *L. plantarum* treatment in intestinal tissues. (a) Serum concentrations of FD4K 4 h after feeding with FD4K by gavage. (b) Serum endotoxin levels. (c) The terminal ileum was utilized to see the transcriptomic changes induced by *L. plantarum* treatment. (d) Principal component analysis plot. (e) Box plot for normalized counts. (f) Heatmap representation of 13 genes belonging to the "establishment of endothelial intestinal barrier" (GO:0090557). (g) Volcano plot depicting overall gene expression change of *L. plantarum* group compared with vehicle

mice. Intestinal barrier function-related genes in (f) were marked in deep blue dots. **P* < 0.05, ** *P* <0.01 compared with the vehicle-treated mice.

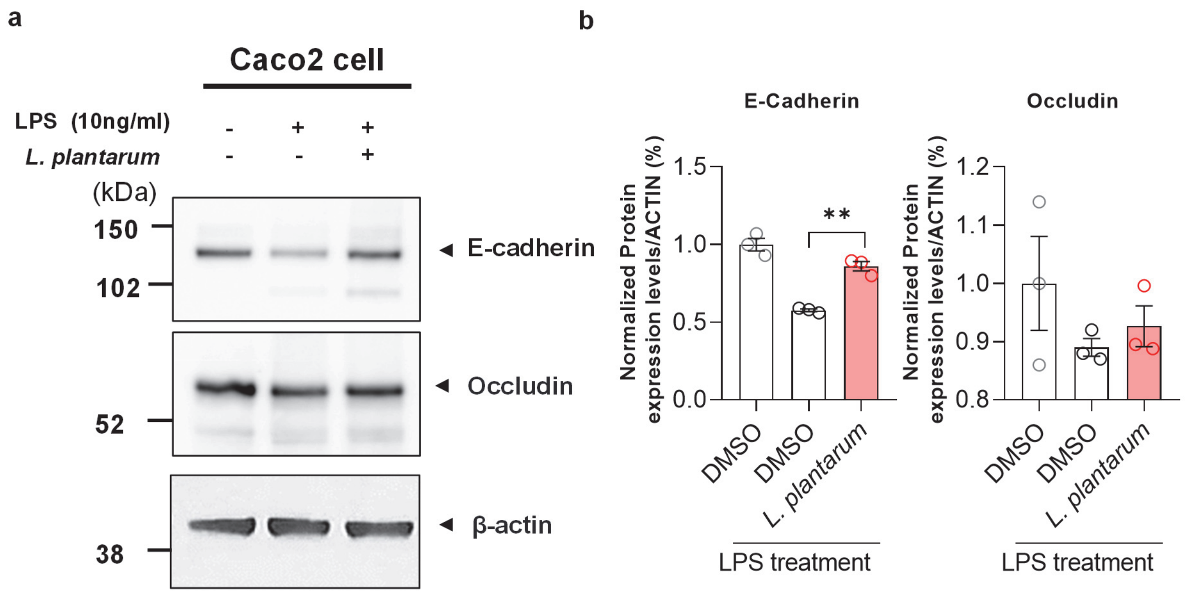

Supplementary Fig. 6. Tight junction protein levels in Caco-2 cells. (a) Representative immunoblot analyses of E-cadherin and occludin. (b) Bar graphs represent band intensities of the blot. Data is the mean ± SD of more than three independent experiments. Band intensities were normalized to β-actin. ** $P < 0.01$. Data points were logarithmically transformed to obtain normality before testing. LPS, lipopolysaccharide.

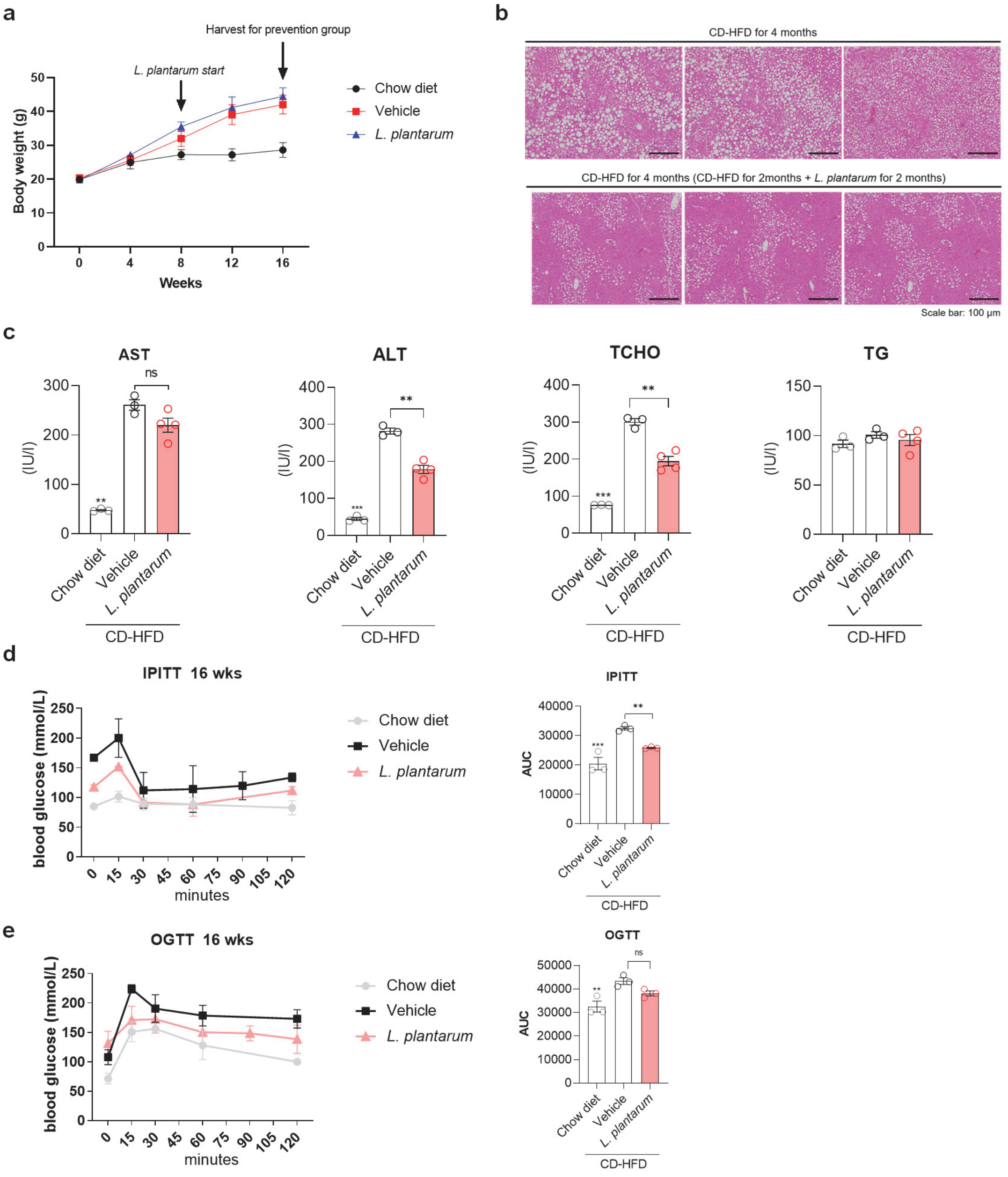

Supplementary Fig. 7. Effect of *L. plantarum* on NAFL murine models. (a) Body weight over time. (b) Representative microphotographs of H&E-stained liver sections. Scale bar = 100 μm. (c) Serum biochemistry levels. (d, e) Comparing OGTT and IPITT at 16 weeks, mice were subsequently fed with CD-HFD for eight weeks and administered L. plantarum for eight weeks. **P* < 0.05, ** *P* <0.01 compared with the CD-HFD vehicle-treated group, using the posthoc Tukey test after ANOVA. CD-HFD, choline-deficient high fat diet; AST, aspartate aminotransferase; ALT, alanine aminotransferase; TCHO, total cholesterol; TG, triglyceride; IPITT, intraperitoneal insulin tolerance test; OGTT, oral glucose tolerance test.

a

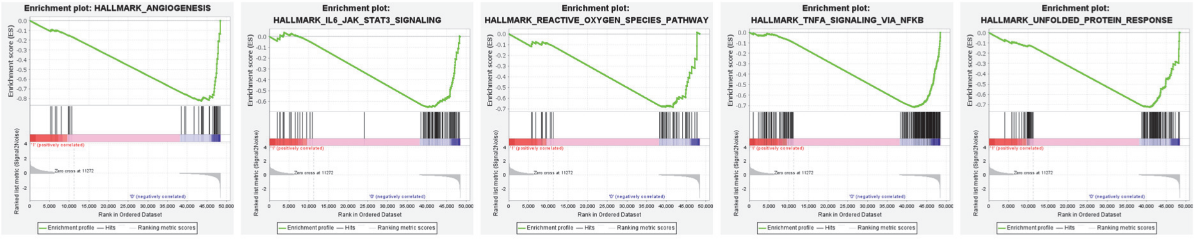

b

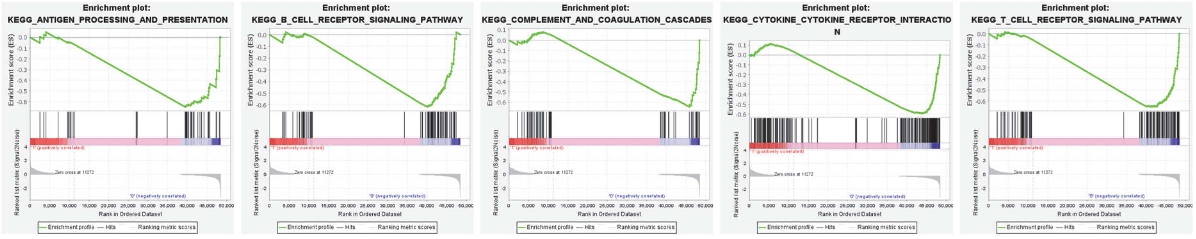

Supplementary Fig. 8. GSEA enrichment plots for the top enriched category in L-arginine treatment related to Figure 6. (a, b) Inflammation-related terms in L-arginine treated group compared with the vehicle group. Based on the Hallmark (a) and the KEGG pathway (b).

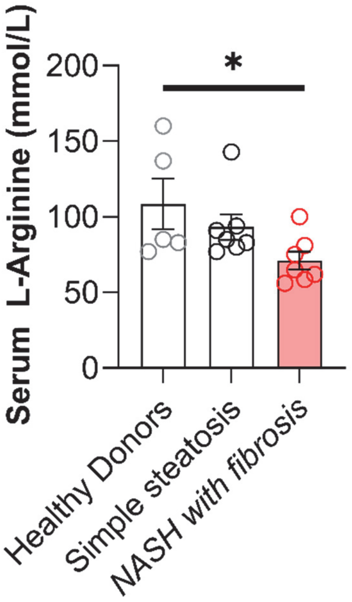

Supplementary Fig. 9. Levels of L-arginine across various stages of NAFLD in human NAFLD. L-arginine levels decreased as NAFLD progressed.

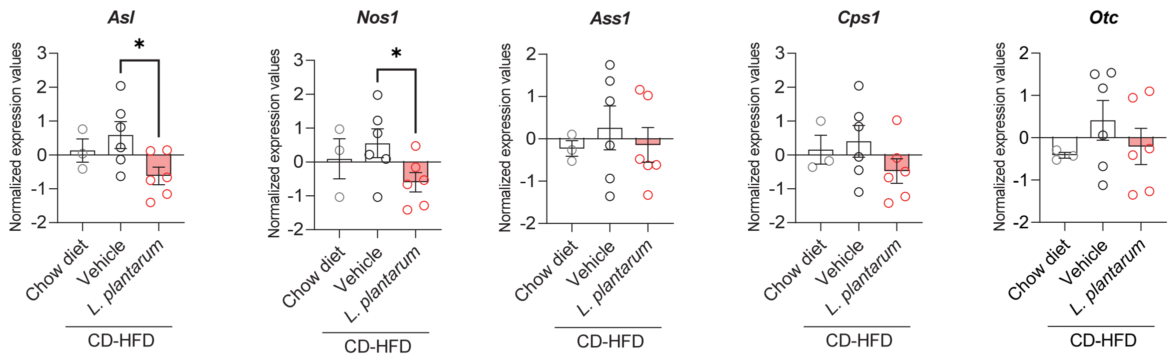

Supplementary Fig. 10. Relative mRNA levels of genes related to L-arginine biosynthesis in liver transcriptomic data.

SUPPLEMENTARY TABLES

Supplementary Table 1. List of antibodies used for Immunohistochemistry analysis and Western blot assay

Name	*Supplier*	*Cat no.*
F4/80	Invitrogen	Cat# MA5-16363
Myeloperoxidase (MPO)	Invitrogen	Cat# PA5-16672
E-cadherin	Cell Signaling Technology	Cat# 3195
Occludin	Novus	Cat# 87402
HNF-4alpha	Santa Cruz Biotechnology	Cat# sc-374229
Albumin	R & D systems	Cat# MAP-1455
β -Actin	Abcam	Cat# ab20272

Supplementary Table 2. Primers used for real-time PCR in this study

Gene	*Species*	*Sequence (5'-3')*
IL-1β _F	Mouse	CCAGCTTCAAATCTCACAGCAG
IL-1β _R	Mouse	CTTCTTTGGGTATTGCTTGGGATC
IL-12_F	Mouse	GAGCACTCCCCATTCCTACT
IL-12_R	Mouse	GCATTGGACTTCGGTAGATG
GAPDH_F	Mouse	ACTCCACTCACGGCAAATTC
GAPDH_R	Mouse	TCTCCATGGTGGTGAAGACA
**Srebf1_F**	Mouse	TGA CCC GGC TAT TCC GTG A
**Srebf1_R**	Mouse	CTG GGC TGA GCA ATA CAG TTC
**Fasn_F**	Mouse	GCG GGT TCG TGA AAC TGA TAA
**Fasn_R**	Mouse	GCA AAA TGG GCC TCC TTG ATA
**Acaca_F**	Mouse	CAG TAA CCT GGT GAA GCT GGA
**Acaca_R**	Mouse	GCC AGA CAT GCT GGA TCT CAT
**Pparg_F**	Mouse	GGT GTG ATC TTA ACT GCC GGA
**Pparg_R**	Mouse	GCC CAA ACC TGA TGG CAT TG
**Gpam_F**	Mouse	CCA CAG AGC TGG GAA AGG TT
**Gpam_R**	Mouse	GTG CCT TGT GTG CGT TTC AT
**Cav3_F**	Mouse	AGC CGC GTC TAC TCC ATC TA
**Cav3_R**	Mouse	TCT CTT TCT GCG TGC TGA TG
**CD36_F**	Mouse	GCA AAG AAG GAA AGC CTG TG
**CD36_R**	Mouse	CCA ATG GTC CCA GTC TCA TT
**Fabp1_F**	Mouse	CAT CCA GAA AGG GAA GGA CA
**Fabp1_R**	Mouse	CCC AGT CAT GGT CTC CAG TT
**Slc27a5_F**	Mouse	TCG GAT CTG GGA ATT CTA CG
**Slc27a5_R**	Mouse	CAG GAA TGC AAA AAC CCT GT
**Acox1_F**	Mouse	CAC GGC TAT TCT CAC AGC AG
**Acox1_R**	Mouse	CAG GCT GTT AAT GTC CAC CA
**PParα_F**	Mouse	ACG ATG CTG TCC TCC TTG ATG
**Pparα_R**	Mouse	GTG TGA TAA AGC CAT TGC CGT

**Acat1_F**	Mouse	CCC CAT TGA TTT TCC ACT TG
**Acat1_R**	Mouse	AGC ACA ACC ACA CTG AAT GC
**Cpt1a_F**	Mouse	CCT GCA TTC CTT CCC ATT TG
**Cpt1a_R**	Mouse	TGC CCA TGT CCT TGT AAT GTG
**Mttp_F**	Mouse	GAA AGA AGT GCT CCC TCA G
**Mttp_R**	Mouse	CCT TTG AAC TTA CTA AGG AGG G
**ApoB_F**	Mouse	TAC TTC CAC CCA CAG TCC CCT
**ApoB_R**	Mouse	CCT TAG AAG CCT TGG GCA CAT

Supplementary Table 3. Basic characteristics of the participants in the human liver transcriptomic cohort

Clinical features	*NAFLD (n = 28)*	*Healthy control (n = 5)*
Age (year)	43.07 ± 15.06	26 ± 5.36
Sex, male (%)	17 (60.7)	5 (100)
DM (%)	11 (39.2)	0
Height (cm)	170.46 ± 8.59	176.53 ± 6.49
Weight (cm)	87.46 ± 20.42	79.12 ± 10.82
BMI (kg/m^2)	29.82 ± 5.21	25.31 ± 2.49
AST (U/L)	76.16 ± 30.61	16.12 ± 3.79
ALT (U/L)	91.25 ± 53.59	15.22 ± 3.65
γ-GTP (U/L)	139.03 ± 79.61	20.23 ± 3.96
Total cholesterol (mg/dL)	188.21 ± 40.01	181.21 ± 38.85
Triglyceride (mg/dL)	195.35 ± 96.24	97.80 ± 25.21
HDL-cholesterol (mg/dL)	43.33 ± 7.52	42.80 ± 9.94
LDL-cholesterol (mg/dL)	127.40 ± 39.57	98.73 ± 43.63
***Liver Histology*†**		
Steatosis	1.85 (1-3)	NA
Inflammation	1.57 (1-3)	NA
Ballooning	1.25 (0-2)	NA
NAFLD Activity Score	4.67 (2-7)	NA

Notes: Data are expressed as number (%) or mean ± standard deviation. †Values for liver histology are expressed as mean (min-max).

Abbreviations: DM, diabetes mellitus; BMI, body mass index; AST, aspartate aminotransferase; ALT, alanine aminotransferase; γ-GTP, γ-glutamyl transferase; HDL, high-density lipoprotein; LDL, low density lipoprotein; NA, not applicable.

Supplementary Table 4. Relative abundance of bacterial composition estimated by genome-based profiling using shotgun sequencing data

Species	*Chow diet*	Vehicle group	*L. plantarum* group
Faecalibaculum_rodentium	*35.47243*	0.635758	4.894642
Muribaculaceae_bacterium_DSM_103720	*14.60647*	1.879778	0.596436
Bacteroides_vulgatus	*14.26879*	56.24696	20.85911
Muribaculum_intestinale	*13.58573*	0.095878	1.900638
Lactobacillus_intestinalis	*3.820192*	0.253843	0
Bacteroides_caccae	*3.278392*	3.728053	1.567714
Lactobacillus_reuteri	*3.204472*	0.38465	0
Bifidobacterium_pseudolongum	*2.450714*	5.072518	0.398562
Lactobacillus_murinus	*1.977508*	1.759658	3.247004
Mucispirillum_schaedleri	*1.777474*	3.83332	29.54553
Enterorhabdus_caecimuris	*1.382062*	0.59501	0.609636
Parabacteroides_goldsteinii	*1.183696*	3.83323	6.924694
Anaerotruncus_sp_G3_2012	*0.883488*	1.218993	1.156412
Lachnospiraceae_bacterium_28_4	*0.432074*	1.361583	8.261528
Lactobacillus_johnsonii	*0.329346*	0.41756	0.625662
Clostridium_sp_ASF502	*0.23002*	0.158865	0.361566
Akkermansia_muciniphila	*0.207396*	12.42145	4.322594
Adlercreutzia_equolifaciens	*0.159166*	0.011948	0.01259
Dorea_sp_5_2	*0.154344*	0.015433	0.108972
Lachnospiraceae_bacterium_A4	*0.141234*	0.34071	0
Lachnospiraceae_bacterium_A2	*0.111222*	0.99498	0.002626
Lachnospiraceae_bacterium_COE1	*0.086364*	0	0
Asaccharobacter_celatus	*0.05564*	0.004845	0.004928
Firmicutes_bacterium_ASF500	*0.051598*	0.109635	0.009174
Oscillibacter_sp_1_3	*0.04001*	0.113228	0.093656
Acutalibacter_muris	*0.03684*	0.071655	0.071282
Clostridium_sp_ASF356	*0.029736*	0	0
Lachnospiraceae_bacterium_3_1	*0.021034*	0.005305	0.002918
Lachnospiraceae_bacterium_M18_1	*0.014164*	0.003703	0.027728
Lachnospiraceae_bacterium_10_1	*0.00416*	1.653088	0.028392
Escherichia_coli	*0.003472*	0.003335	0.30838
Clostridium_cocleatum	*0.000544*	0.374628	0
Eubacterium_plexicaudatum	*0.000126*	0.000393	0.028072
Eubacterium_sp_14_2	*0.000102*	0.021123	0.013282
Bacteroides_caecimuris	*0*	0	0.980864
Bacteroides_sartorii	*0*	0	0.031734
Bacteroides_uniformis	*0*	0	0.085286
Prevotella_sp_MGM1	*0*	0.01246	0
Prevotella_sp_MGM2	*0*	0	1.10701
Parabacteroides_distasonis	*0*	0.070713	0.392228
Parabacteroides_merdae	*0*	0	0.001358
Staphylococcus_aureus	*0*	0	0.008852
Staphylococcus_sciuri	*0*	0.000253	0.008886
Enterococcus_faecalis	*0*	0.00489	0.288056
Lactobacillus_plantarum	*0*	0.018343	9.655816
Lactococcus_lactis	*0*	1.660193	0.85178
Acetatifactor_muris	*0*	0.169973	0.074428
Lachnospiraceae_bacterium_3_2	*0*	0.271855	0.19921
Romboutsia_ilealis	*0*	0.116015	0.104724

Anaerotruncus_colihominis	*0*	0.001098	0.005262
Ruthenibacterium_lactatiformans	*0*	0.00275	0
Ileibacterium_valens	*0*	0.033863	0.021378
Proteus_hauseri	*0*	0.016508	0.070356
Proteus_penneri	*0*	0	0.129058
